# Phosphate Recovery from Swine Wastewater by a Struvite Precipitation Electrolyzer

**DOI:** 10.1038/s41598-019-45085-3

**Published:** 2019-06-20

**Authors:** Fang Wang, Rao Fu, Hang Lv, Guoliang Zhu, Binwei Lu, Zheng Zhou, Xu Wu, Huanchun Chen

**Affiliations:** 1 School of Environmental Science and Engineering, Huazhong Unviersity of Science and Technology, 1037 Luoyu Road, Wuhan, 430074 China; 2 Hubei Meichen Environmental Protection Science and Technology Co., Ltd., No. 6 Gaoxin Road, High-tech Zone, Jingmen, 448000 China; 3 College of Animal Science and Technology, Huazhong Agricultural University, 1 Lion Rock, Wuhan, 430070 China

**Keywords:** Pollution remediation, Chemical engineering

## Abstract

Struvite precipitation electrolyzers are interesting environmental electrochemical reactors with potential applications for efficient phosphate recovery from wastewater, such as swine wastewater. In this paper, effects of phosphate concentration and pH on the struvite precipitation reaction rate were investigated. When phosphate concentration decreased from 100 to 20 mg/L, the precipitation reaction rate decreased from 396.65 mg/L·h to 70.46 mg/L·h, indicating that the reaction rate of struvite crystallization can be controlled by adjusting pH according to the change of phosphate concentration. Numerical simulation of different currents and flow rates on pH in the electrolyzer was developed and validated, and pH in the electrolyzer was dynamically measured along the distribution point of the flow field. We aimed to test the treatment effect of the electrolyzer on actual swine wastewater. When the flow rate was 20 L/h and constant voltage was 4 V, the electrolyzer was run continuously for 5 hours with the volume of 50 L. The phosphate recovery efficiency reached 99.51%, and the time-space yield of the struvite precipitation electrolyzer was 0.0219 kg/m2·h. The harvested struvite particles were identified by XRD and SEM-EDS, which presented orthorhombic structure and high purity. Economic analysis demonstrated that the proposed electrolyzer was cost-effective and technologically convenient.

## Introduction

Electrochemical; the struvite precipitation electrolyzer; the reaction rate; regulation of pH; phosphate recovery.

China Statistical Yearbook of 2018 showed that 70202.1 million pigs were sold in that year. Pig breeding plays an important role in China’s agriculture. Raising pigs can solve the problem of the supply of livestock products and drive the rural economic development, bringing about serious environmental pollution problems as well. In recent years, the pollution of livestock and poultry industry has become more and more serious, and the total phosphorus (TP) concentration in swine wastewater can reach 100–1400 mg/L^1^. Therefore, it is an urgent task to recover nutrient by technical means.

The recovery of phosphorus in the form of struvite precipitation has been recognized by many domestic and foreign experts as one of the most promising technical methods. This method can not merely reduce the environmental pollution caused by phosphorus emission, but also can be used in the fertilizer industry^2^. The struvite precipitation consists of magnesium, ammonium, and phosphate with equal molar concentration^3^.

Its reaction can be expressed as the following formula^4–7^:

1 $${{\rm{Mg}}}^{2+}+{{\rm{NH}}}_{4}^{+}+{{{\rm{HPO}}}_{4}}^{2-}+6{{\rm{H}}}_{2}{\rm{O}}\to {{\rm{MgNH}}}_{4}{{\rm{PO}}}_{4}\,\cdot \,6{{\rm{H}}}_{2}{\rm{O}}+{{\rm{H}}}^{+}$$

The solubility product of struvite (K_sp_) is 10^−13.26^^ 8^, and swine wastewater contains high concentration of nitrogen and phosphorus^9^. Therefore, struvite precipitation can achieve better results in the treatment of nitrogen and phosphorus.

In recent years, there is a lot of literature on swine wastewater by using struvite precipitation^10–12^. In some literature, the struvite crystallization process and the recovery efficiency of the phosphate depended on concentration of phosphate, ammonium and magnesium ions, pH, the flow rate, and the N/P ratio^13–15^. Liu *et al*.^16^ reported that K^+^ and Ca^2+^ in the swine wastewater can influence the formation of struvite crystals. Through experimental study, Anna KOZIK *et al*.^17^ pointed out that the presence of organic impurities may affect the final size and shape of struvite crystals. Hao *et al*.^18^ indicated that when the pH range was 7.5–9.5, the yield of precipitated products was the largest. Uludag-Demirer and Othman^19^ found that with the supernatant of anaerobically digested waste activated sludge became more saturated in terms of struvite forming ions, the effect of pH increased became insignificant in the removal of ions from the solution. Song *et al*.^20^ revealed that utilizing the method of struvite precipitation for simulated swine wastewater was effective and the Ca ion effectively inhibited the formation and purity of struvite. Huang *et al*.^21^ reported that the phosphorus in swine wastewater was recovered efficiently in the form of struvite precipitation by magnesium metal corrosion. Through the comparison of the above literatures, it is not difficult to find that recovery of phosphate from livestock wastewater by struvite crystallization has been studied extensively. However, at present, the most common approach of struvite precipitation from swine wastewater is chemical precipitation by adding magnesium salts and adjusting pH with the added agent^22,23^. In order to reduce the cost of phosphate recovery, some researchers use the magnesium like MgCO_3_ and Mg (OH)_2_ as the magnesium source, but those reagents are considered to decrease the purity of the struvite^24,25^. Hug and Udert^26^ used electrolytic magnesium dissolution for swine wastewater by means of struvite precipitation, proving that the average phosphate removal rate was 3.7 mg P cm^−2^·h^−1^, with current density of 5.5 mA·cm^−2^ and current efficiency of 118%. Additionally, Moussa *et al*.^27^ also found that the electrochemical methods can obtain the pure struvite production. But by building a new type of electrochemical struvite precipitation of phosphorus recovery unit (especially magnesium board is used as the electrode), and Comsol software was used to simulate device performance and optimize reaction conditions, so as to obtain feasible process route and parameters. Compared with other methods (such as medicament method), this method has the advantages of high efficiency, low cost, low energy consumption and strong applicability. Consequently, the use of the electrochemical reactor may be an interesting process for phosphate recovery from sewage by struvite precipitation, which especially improves the time-space yield of the struvite precipitation electrolyzer.

The objective of this study was to improve the time-space yield of the struvite precipitation electrolyzer by controlling the pH. First, the *in-situ* ultraviolet visible (UV-vis) spectroscope was adopted to determine the struvite precipitation reaction rates with different phosphate concentration and pH. Scanning electron microscope-energy dispersive spectrometer (SEM-EDS) pictures, particle size distribution and X-ray diffraction (XRD) patterns of the struvite precipitation at different reaction rates were observed. Second, a finite element multiphysics simulation model was established (with Comsol Software), and a series of experiments were conducted at different flow rates and currents to validate the model. Third, based on the aforementioned results, a continuous-flow experiment was tested for phosphate recovery from actual swine wastewater by the struvite precipitation electrolyzer. Finally, an economic analysis was evaluated.

## Results and Discussion

### Effect of different phosphate concentration and pH value on the struvite precipitation reaction rate

As expressed by Eq. (1), the reaction formation process of struvite is related to the simultaneous presence of Mg^2+^, NH_4_^+^ and HPO_4_^2−^ in the sedimentation. It can be seen from the formula that pH is an important indicator for struvite formation.

As is shown in Eq. (2) and Eq. (3), Mg in the anode is oxidized to Mg^2+^ in the experiment, generating two electrons. At the same time, in the cathode, H_2_O is reduced to H_2_, and the producing of OH^−^ increases the pH. With the increase of OH^−^ concentration, the crystallization conditions are formed gradually. The struvite precipitation would be produced by the sacrificial magnesium anode in the swine wastewater, which has high concentration of PO_4_^3−^ and NH_4_^+^.

2 $${\rm{Mg}}\to {{\rm{Mg}}}^{2+}+2{{\rm{e}}}^{-}$$

3 $$2{{\rm{H}}}_{2}{\rm{O}}+2{{\rm{e}}}^{-}\to {{\rm{H}}}_{2}\uparrow +2{{\rm{OH}}}^{-}$$

**Figure 1:**
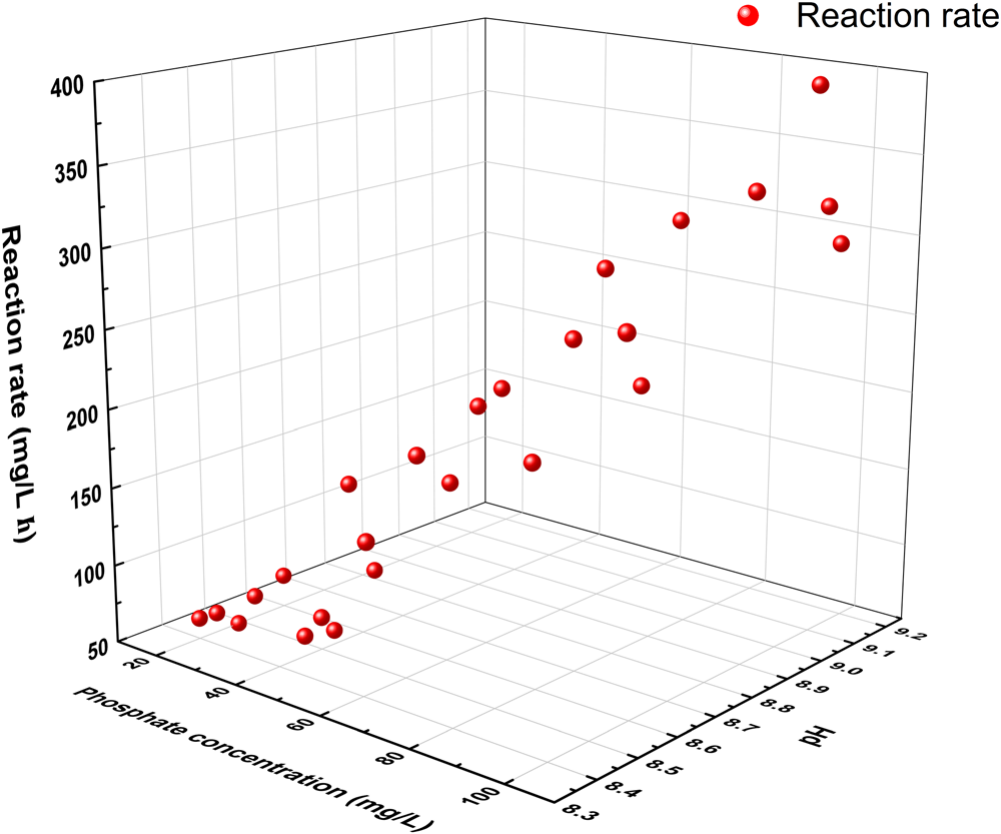
Change of the struvite precipitation reaction rate under different phosphate concentration and pH conditions.

As can be seen from the paragraph above, solution pH is the crucial factor that influences the struvite crystallization. However, the reaction rate represents the rate of the struvite precipitation. To investigate the impact of different ${{{\rm{P}}{\rm{O}}}_{4}}^{3-}{\textstyle \text{-}}{\rm{P}}$ concentration and pH on the reaction rate of struvite precipitation, a series of *in-situ* UV-vis experiments have been carried out. The results were shown in Fig. 1. Figure 1 indicated that the phosphate concentration and solution pH have an important influence on the reaction rate in the process of struvite precipitation. With an increasing phosphate concentration and solution pH, the reaction rate increased rapidly. When the phosphate concentration increased from 20 to 100 mg/L, and solution pH increased from to 8.6 to 9.08, the reaction rate incereased from 70.46 to 396.65 mg·L^−1^·h^−1^. This reslut shows that the reaction rate of struvite crystallization can be controlled by adjusting pH according to the change of phosphate concentration. As reported by Huang *et al*.^28^, the recovery rate of phosphate increased with the increase of pH, and too high pH will affect the purity of struvite. Huang *et al*.^29^ reported that when the solution pH increased from 8.5 to 12.0, the removal efficiency of PO_4_^3-^-P rose from 64% to 78%, and as the solution pH further increased from 12.0 to 12.5, the PO_4_^3-^-P removal efficiency rapidly declined. However, the pH was in the range 5.0 to 9.0, TP removal efficiencies were all above 70%^30^.

SEM-EDS pictures, particle size distribution and XRD patterns of the struvite precipitation of different reaction rates were demonstrated in SI Figs S1–S5, showing that the particle size with the highest reaction rate was the smallest and the deviation of relative frequency of the particle size distribution was near 6 *μ*m. The phenomenon can be explained that the over-saturation of the struvite crystal reaction went up with the increase of pH value of the reaction system and the concentration of phosphate. It was not conducive to the growth and the aggregation of the struvite crystals, resulting in the reduction of the particle size of the recycled products. This result was consistent with the results of other literature studies^31,32^. As can be seen from SEM-EDS pictures, with the increase of the reaction rate, the shape of the precipitate also changed significantly, from the rhumb to the slender needle shape. According to the XRD analysis results, the main diffraction peak of the crystal is dominated by the characteristic peak of magnesium ammonium phosphate, indicating that the main component of the precipitate is struvite.

In this paper, the study of the reaction rate can not only control the reaction rate of struvite precipitation by controlling the pH, but also provide support for the establishment of the device model.

### Effect of different currents and flow rates on pH value in the electrolyzer

#### Numerical simulation

**Figure 2:**
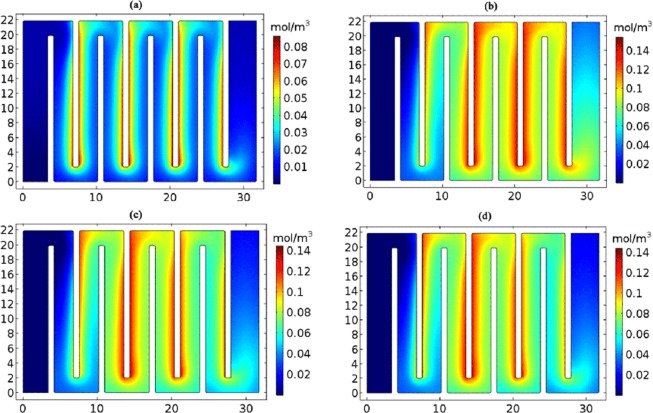
The model diagrams of variation and distribution of pH in the electrolyzer at the reaction time of (**a**) 100 s, (**b**) 1000 s, (**c**) 2500 s and (**d**) 3700 s. (The flow rate, 20 L/h; the current, 1.13 A).

Figure 2 demonstrated the simulation results for the variation and distribution of pH in the electrolyzer, with the flow rate of 20 L/h and the current of 1.13 A. As showed in Fig. 2a, when the reaction time was 100 s, a large number of OH ions were produced on the cathode surface and began to diffuse outward; as observed in Fig. 2b, when the reaction time was 1000 s, OH ions in the downstream of the flow channel had been completely diffused into the whole flow channel and pushed to the outlet. But in the upstream, due to the inflow of the reactants Mg, NH_4_ and HPO_4_, the struvite was formed and protons were produced. At this time, some of the OH ions would be consumed because of the neutralization reaction, but new OH ions were still produced on the surface of the electrode; as exhibited in Fig. 2c, in 2500 seconds, the reactants had flowed from the upper reaches to the lower reaches. By this stage, the precipitation reaction, the electrode interface reaction and the neutralization reaction had reached dynamic equilibrium in the entire flow channel; as Fig. 2d showed, when the reaction time reached 3700 s, the reactant concentration distribution of the entire flow channel reached a stable state. Numerical simulation results for the variation and distribution of pH at different flow rates and currents were presented in SI Figs S6–S13.

**Figure 3:**
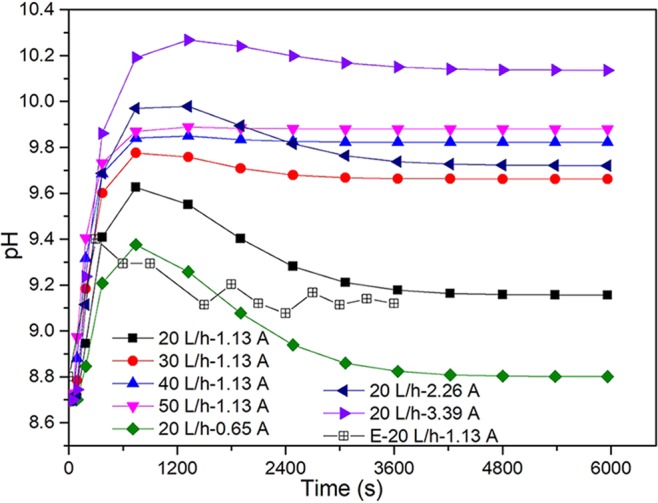
The variation graph of pH value at the outlet of the electrolyzer under different flow rate and current conditions. (E represents pH values of experiment measurement, and the rest is pH values of numerical simulation).

Figure 3 displayed the pH value variation graph of numerical simulation and experimental measurement at the outlet of the electrolyzer. It can be seen from the figure that pH value at the outlet of the electrolyzer showed a trend of first increasing and then decreasing under different current and flow rate conditions with the reaction time. When the reaction time was around 600 s, the pH of the numerical simulation was the highest, which decreased gradually. Nevertheless, the pH of the experimental measurement was the highest when the reaction time reached 300 s. In the process of numerical simulation, other factors such as the consumption of partial OH ions by other reactions in the electrolyzer were ignored. Therefore, controlling different currents and flow rates can not only fully regulate pH, but also provide guidance and reference for further experimental studies on different currents and flow rates.

#### Experimental measurement

To verify the different current and flow rate on pH in the electrolyzer, the dynamical variation and distribution of pH in the electrolyzer were observed by experimental research. As shown in experimental setup, the distribution points of 5 flow fields in the electrolyzer were taken as the research object and measured every 5 minutes. The distribution of pH with time in combination with different flow rates and currents was studied in an hour, and the results were shown in S1 Figs S14–S19. It was observed from S14–S19 that the current and flow rate exerted a significant influence on the pH of the electrolyzer. For example, in the first channel, the pH increased with the increase of the current at different flow rates at an identical reaction time, but the growth was slow. As the first channel was the inlet of the electrolyzer, the hydrogen evolution reaction did not completely occur by this time when the solution entered the electrolyzer. In the second, third and fourth channels, the pH changed quite dramatically under the same condition. As the current rose, the pH increased rapidly. The reasons may be that the magnesium plate anode released a large number of magnesium ions with the increase of the current, and the cathode also underwent the hydrogen evolution reaction which increased the pH. Compared with the second, third and fourth channels, the pH of the fifth channel was relatively low and grew slowly, which was shown in formula 1. When the precipitation reaction of struvite occurred, hydrogen ions were produced simultaneously. However, because of the neutralization reaction, the hydroxyl ions in the solution were consumed. Thus, the pH decreased.

Through the numerical model and experimental research, we can find that according to the pH conditions of struvite precipitation, the study on the pH dynamic distribution and variation over time in the distribution point of the flow field in the electrolyzer can help improve the time-space field of the struvite precipitation electrolyzer by controlling pH under certain current and flow rate conditions.

### Treatment of actual swine wastewater

**Figure 4:**
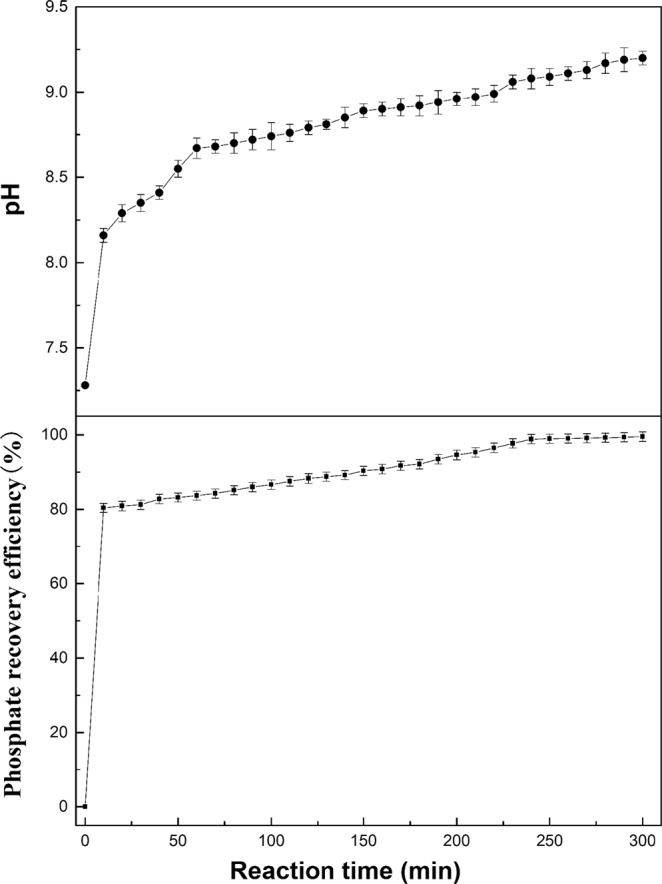
The change of phosphate recovery efficiency and the solution pH over the time.

**Figure 5:**
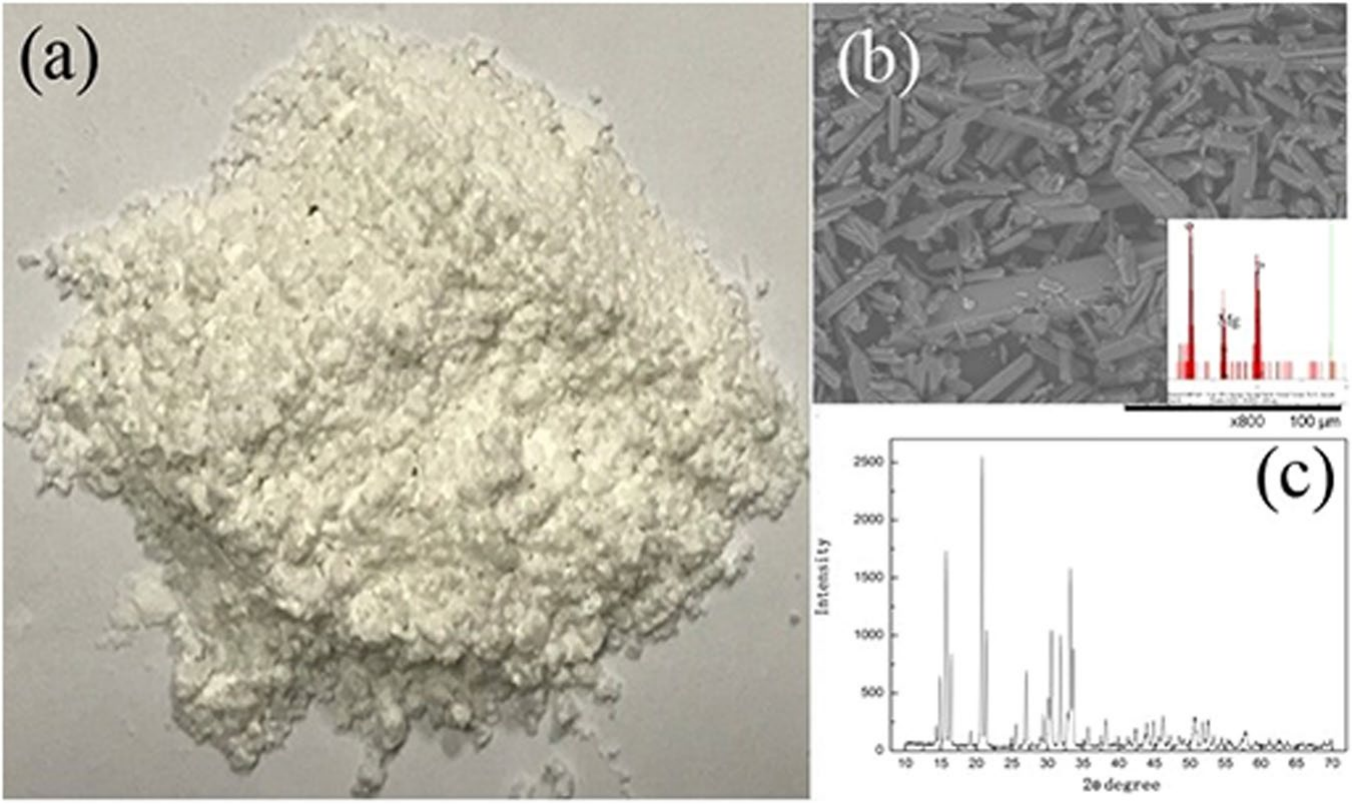
The picture (**a**), SEM-EDS micrograph (**b**) and XRD patterns (**c**) of the struvite collected from actual swine wastewater.

Based on the above research, we aimed to investigate the treatment of the actual swine wastewater by the electrolyzer, and the experiment was conducted with the following conditions: the feed volume of 50 L; reaction time of 300 min; and the feed flow rate of 20 L/h. The feed solution was the actual swine wastewater. Because the composition of actual swine wastewater was more intricate, the constant voltage of 4 V was conducted. The experimental results were exhibited in Fig. 4, indicating that the phosphate recovery efficiency rapidly increased to 83.66% in the initial 60 min and gradually increased to 99.51%. The corresponding pH increased from 7.28 to 8.67, and then increased to 9.2. Under the condition of constant voltage, the Mg metal plate was oxidized to Mg^2+^ enormously in the anode. However, in the cathode, OH^−^ was generated simultaneously so that the pH rose. Furthermore, the concentration ratio of PO_4_^3−^-P: NH_4_^+^-N was 1:2 in actual swine wastewater. At the same time, the feed flow rate was set to 20 L/h, and the struvite precipitation was formed rapidly under this condition. Consequently, the phosphate recovery efficiency was enhanced, which testified that recovering the phosphate from actual swine wastewater by the electrolyzer was effective and feasible. In addition, the struvite precipitation collected from the collection tank (Fig. 5a) was air dried and then observed by SEM-EDS and XRD. The SEM-EDS picture (Fig. 5b) exhibited that the morphology of the reaction product was the needle-like orthorhombic crystal. The XRD diffractogram (Fig. 5c) indicated that the component of the reaction product was struvite.

### Economic analysis

In this paper, the economic analysis was carried out for the recovery of phosphate from actual swine wastewater by the electrolyzer. The above results show that the mass concentration of phosphate decreased from 136.3 mg/L to 0.66 mg/L, and this data have satisfied the China standards for effluent discharge of livestock and poultry. Moreover, we obtained struvite of 17.52 g, and the time-space yield of struvite precipitation was 0.0219 kg/m^2^·h. The energy consumption was presented in S1 Fig. S20, revealing that the energy consumption (Q) of the experimental system run for 5 hours was 2.033 kWh/ton. Table 1 listed the market price and operation costs of total items, and compared it with other methods. Apart from the cost of the magnesium source and other chemicals used in this economic evaluation, it is assumed that the cost of devices (pump operation, labor, maintenance and installation, etc.) were the same. The total operation cost of the electrolyzer was 0.07246 ¥/50 L, namely 1.45 ¥/m^3^. However, the total operation cost of the reagent method was 5.1 ¥/m^3^ in published literature^1^. Xiao *et al*.^1^ claimed that the cost recovery of phosphate from swine wastewater using the supernatant of the waste sulfuric acid after dolomite neutralization was 1.7 ¥/m^3^, indicating that their total operation costs were higher than ours. In addition, Huang *et al*.^21^ also reported that the recovery cost of phosphate from swine wastewater by magnesium metal corrosion was 1.43 ¥/m^3^, demonstrating that there was no obvious difference in the total operation cost of our two methods. When comparing our results with those studies, the electrolyzer can not only recover phosphorus efficiently without adding reagents, but also produce struvite to reduce the operation cost of swine wastewater. Therefore, the proposed electrolyzer is effective and feasible, and the device is simple, stable and controllable.

**Table 1:** Theoretical economic analysis of our electrolyzer method compared with other methods.

Item	Our electrolyzer		Mg metal corrosion		Reagent method		Other method	
	Market price (¥/kg)	Dosage (kg or kWh)	Market price (¥/kg)	Dosage (kg)	Market price (¥/kg)	Dosage (kg)	Market price (¥/kg)	Dosage (kg)
Mg metal (99.9%) (¥)	10	−0.00169	−3.43		—	—	—	—
Energy consumption (Q) (¥/kWh)	0.65	−0.10165	—	—	−2.1		−3.7	
Struvite (¥)	0.6	+0.01752	+2		+2		+2	
Phosphate recovery	99.51%		97.5%		99%		95%	
Reagent cost (¥) (contains some magnesium salt and NaOH, etc.)	—	—	—	—	−5		—	—
Total operation cost (¥/m^3^)	−1.45		−1.43		−5.1		−1.7	

Note: “+” represents income, “−” represents invest.

## Conclusions

Through *in-situ* UV-vis analysis, we found that when phosphate concentration decreased from 100 mg/L to 20 mg/L, the precipitation reaction rate decreased from 396.65 mg/L·h to 70.46 mg/L·h, which exhibited that the reaction rate of struvite crystallization can be controlled by adjusting pH according to the change of phosphate concentration. Based on the reaction rate obtained from experiment, a model for the electrolyzer has been developed and used to simulate the pH kinetic change process along the distribution point of the flow field in the electrolyzer at different flow rates and currents. Based on experimental validation of the model parameters, the model was in good agreement with the experimental pH under the condition of the flow rate and the current. In accordance with the above research, a continuous-flow experiment using the electrolyzer was established for phosphate recovery from actual swine wastewater. The phosphate efficiency was 99%; the time-space yield of the struvite precipitation electrolyzer was 0.0219 kg·m^−2^·h^−1^; and the total operation cost of our electrolyzer was 1.45 ¥/m^3^. The struvite was identified and analyzed by XRD and SEM-EDS. The economic analysis demonstrated that our electrolyzer can not only recover phosphorus efficiently, but also produce struvite to reduce the operation cost of swine wastewater, especially improving the time-space yield of the struvite precipitation electrolyzer by controlling pH.

## Materials and Methods

### Materials

In the experiments, we prepared simulated swine wastewater with the same concentration of ammonia nitrogen and phosphate as the real wastewater by dissolving analytical grade NH_4_Cl and Na_3_PO_4_·12H_2_O in pure water. The real swine wastewater was obtained from the effluent from anaerobic digest in Lixin pig factory located in Jiangxia District, Wuhan City, Hubei Province, China. This real swine wastewater was filtered to remove some large particles by the 1-µm filter bag prior to use. The characteristics of the pretreated swine wastewater are listed in Table 2.

**Table 2:** Characteristics of the pretreated swine wastewater.

Parameter	Values	Unit
pH	7.28 ± 0.01	—
TOC	640 ± 120	mg/L
COD	2020 ± 400	mg/L
NH_4_-N	282.12 ± 100	mg/L
PO_4_-P	136.3 ± 10	mg/L
TP	176.67 ± 10	mg/L
Mg	18.65 ± 0.5	mg/L
Ca	106.15 ± 0.5	mg/L
Fe	5.9 ± 0.5	mg/L

### Experimental procedures

**Figure 6:**
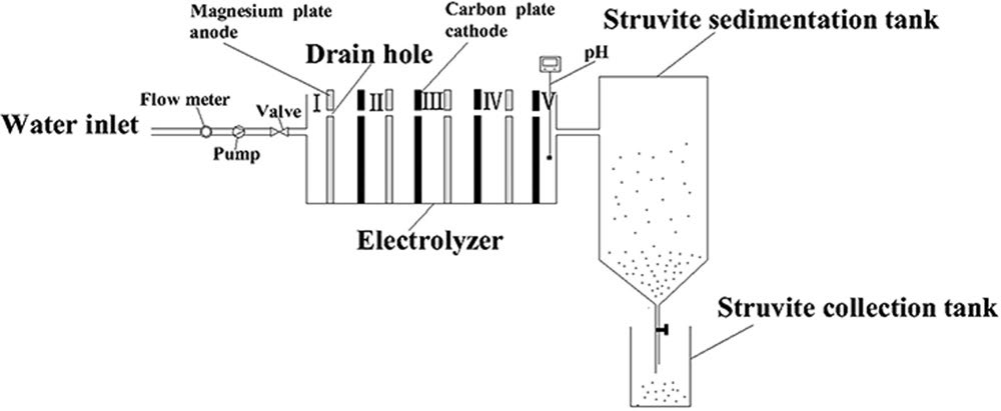
The schematic illustration of experiment set-up.

A schematic diagram of the experimental system is shown in Fig. 6. The electrolyzer consisted of four magnesium plates and four carbon plates, with the magnesium plate being used as the anode and the carbon plate as the cathode. We set 5 flow filed observation points in the electrolyzer; the area of the magnesium plate and the carbon plate was both 0.04 m^2^; the distance between the electrodes was 3.2 cm; and there were some drain holes in the electrodes. The voltage was output by the electrochemical workstation (CS150; Wuhan Corrtest Instrument Co. Ltd; China), and the pH meter was used to monitor the outlet solution pH of the electrolyzer.

The specific experimental procedure contained the following steps. 1) The simulated or pretreated swine wastewater of 50 L was pumped into the electrolyzer and electrolyzed there. 2) The solution after electrolysis flowed into the struvite sedimentation tank. With time passing, the struvite formed and precipitated subsequently, and then was stored in the struvite collection tank and dried in a ventilated place. 3) The treated water was mixed with the raw solution. Subsequently, it was electrolyzed again in the electrolyzer until phosphate concentration of the inlet solution reached the discharge standard.

Besides, in order to determine the effect of different phosphate concentration and pH value on the struvite precipitation reaction rate, a batch of experiments was firstly performed. The experimental conditions were as follows. Concentration of phosphate was 100 mg/L, 80 mg/L, 60 mg/L, 40 mg/Land 20 mg/L, respectively. NH_4_^+^-N concentration was 100 mg/L and Mg^2+^ concentration was 100 mg/L; the rotate speed was 20 rpm and the reaction time was 15 min.

### Analytical methods

A digital pH meter (SW 730; China) was used to measure the pH values. The Inductively Coupled Plasma (OPTIMA 8300DV; America) was utilized to measure the concentrations of Mg^2+^, Ca^2+^ and Fe^2+^ ions in the swine wastewater. The basic potassium permanganate method was applied to the determination of the chemical oxygen demand (COD). A total organic carbon (TOC) analyzer (Multi N/C2100; Germany) was employed to determine the TOC. The concentration of total phosphorus (T-P) and orthophosphate (PO_4_-P) was determined by the phosphomolybdenum blue spectrophotometric method, which used an ultraviolet spectrophotometer (UV5800H; China). The sodium reagent spectrophotometry as well as the ultraviolet spectrophotometer was used to test the concentration of ammonia-nitrogen (NH_4_-N). The morphology of the dried struvite powder was recorded by a scanning electron microscope-energy dispersive spectrometer (SEM-EDS; TM3030; Japan), and the composition was analyzed by the X-ray diffraction (XRD; DX-27mini; China).

### Calculation

In this experiment, the struvite precipitation reaction rate is calculated according to the following formula (4):

4 $$k(mg/L\cdot h)=\frac{C-{C}_{1}}{t}$$

where $k$ is the struvite precipitation reaction rate, and $C$ is the initial mass concentration of phosphate after a while (mg/L). ${c}_{1}$ is the residual mass concentration of phosphate after a period of time (mg/L), and $t$ is the reaction time.

Phosphate recovery efficiency is calculated in accordance with the following formula (5):

5 $$\eta ( \% )=\frac{\rho -{\rho }_{1}}{\rho }$$

where $\eta$ is the recovery efficiency of phosphate, $\rho$ is the initial mass concentration of phosphate (mg/L), and $\,{\rho }_{1}$ is the mass concentration of residual phosphate (mg/L).

Energy consumption ($Q$) in the electrolytic process was estimated by the following equation Eq. (6):

6 $$Q=\int U\cdot Idst$$

where *U* is the applied constant voltage and *I* is the electric current.

### Modeling development

**Figure 7:**
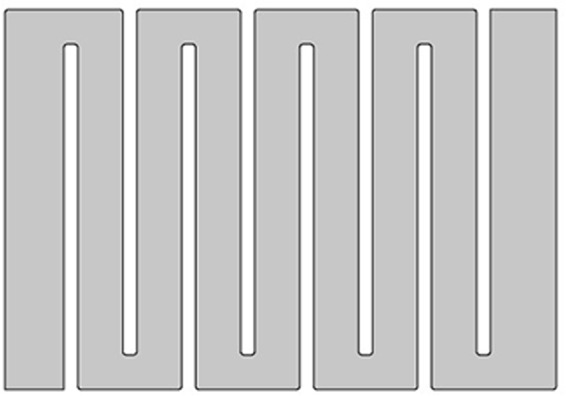
The fluid flow and the reaction area in the electrolyzer.

The model of the electrolyzer is set up by Comsol Multiphysics simulation software (9401017). Figure 7 shows the fluid flow and the reaction area in the electrolyzer. The “Navier-Stokes” equation (N-S equation) of the laminar flow interface is used to calculate the fluid flow:

7 $$\rho u\cdot \nabla u=-\,\nabla p+\nabla \cdot \mu (\nabla u+{(\nabla u)}^{T})$$

8 $$\nabla \cdot u=0$$

where $\rho$ is density (kg/m^3^), and $u$ is velocity (m/s). Besides, $\mu$ is viscosity (N·s/m^2^), and $\,p$ is pressure (Pa). Water is used as the simulated fluid, with the viscosity being 1 × 10^−3^ N·s/m^2^ and the density being 1000 kg/m^3^. At the entrance, we assume that there is a fully developed laminar flow in the model. The speed is set as the parabola distribution, and the average speed is $\,{u}_{inlet}$. At the exit, the pressure of the model is set to zero.

The total mass balance in the electrolyte can be expressed as:

9 $$\nabla \cdot {N}_{i}=0$$

where ${N}_{i}$ is the flux of substance i (the unit of SI is mol·m^2^/s). Its control equation is:

10 $$-{D}_{i}\nabla {c}_{i}-{Z}_{i}{m}_{i}F{c}_{i}\nabla \varnothing +{c}_{i}{u}_{1}={N}_{i}$$

where ${D}_{i}$ is the diffusion coefficient, and ${c}_{i}$ is the concentration of the ion $i$ (the unit of SI is mol/m^3^). ${Z}_{i}$ is valence, and ${m}_{i}$ is the migration rate (the unit of SI is mol·m^2^ (s·V·A)). $F$ is the faraday constant (the unit of SI is C/mol). $\varnothing$ is the potential. ${u}_{1}$ is the velocity vector (the unit of SI is mol·m/s).

The net current density can be described as:

11 $${\rm{i}}=-\,F\,{\rm{\Sigma }}\,{z}_{i}{N}_{i}$$

The current density conservation is:

12 $$\nabla \cdot {\rm{i}}=0$$

The mass transfer of the reaction material is considered according to diffusion, migration and convection, and the resulting mass balance is:

13 $$\nabla \cdot (\,-\,D\nabla C-zmFC\nabla \varnothing +Cu)=0$$

where $D$ is the diffusion coefficient and $C$ is concentration (mol/m^3^).

For the net charge transfer of ions, it is assumed that the model is electrically neutral and it ignores the concentration gradient of the supporting electrolyte, indicating that the potential distribution in the electrolyte can be described by Ohm’s law:

14 $$\nabla \cdot (\,-\,{\rm{\kappa }}\nabla \varnothing )=0$$

where $\kappa$ is conductivity.

The over potential equations of cathode (${\eta }_{c}$) and anode (${\eta }_{a}$) are:

15 $${\eta }_{c}={\varphi }_{l,c}-{E}_{eq,c}$$

16 $${\eta }_{a}={\varphi }_{l,a}-{E}_{eq,a}$$

where ${E}_{eq,c}$ is the cathode equilibrium potential, and ${E}_{eq,a}$ is the anode equilibrium potential.

Flux free boundary conditions are used for all boundary conditions except the inlet, the outlet, the cathode and the anode. The concentration of the electrolyte at the inlet is a fixed value and the outflow conditions apply to the outlet. Faraday is utilized to specify the net molar flux of material consumption on the cathode and the anode:

17 $$-{\rm{n}}\cdot {{\rm{N}}}_{i}={}_{m}{}^{{\rm{\Sigma }}}R_{i,m}$$

18 $${R}_{i}=\frac{{v}_{i}i}{nF}$$

where ${R}_{i}$ is the reaction moles of the material, and $F$ is the faraday constant. $n$ is the number of electrons involved in the reaction, and ${v}_{i}$ is the chemical reaction equivalent coefficient. For the anode reaction, the magnesium electrode is oxidized (n = 2; ${v}_{i}\,$= −1). For the cathode reaction, the proton is reduced (n = 2; ${v}_{i}\,$= 2).

The precipitation reaction of ammonium magnesium phosphate is:

19 $$M{g}^{2+}+N{H}_{4}^{+}+HP{O}_{4}^{2-}+6{H}_{2}{\rm{O}}\to MgN{H}_{4}P{O}_{4}\,\cdot \,6{H}_{2}{\rm{O}}+{H}^{+}$$

20 $${H}^{+}+O{H}^{-}\to {H}_{2}O$$

The pH value is calculated by the following formulate:

21 $${\rm{pH}}=14+l{g}^{{c}_{OH}}$$

Based on the above model settings, the variation and distribution of reactant concentration can be calculated and simulated under the given conditions of inlet velocity and the total current. All parameters in the model are shown in Table 3.

**Table 3:** Parameter setting in the model.

Parameter	Value	description
D__Mg_^33^	1*10^−9^ m^2^/s	Magnesium ion diffusion coefficient
D__OH_	2*10^−6^ m^2^/s	Hydroxide ion diffusion coefficient
C_0_Mg_	0.78 mol/m^3^	The initial concentration of magnesium ion
C_0_OH_	5.012*10^−3^ mol/m^3^	The initial concentration of hydroxide ion
C_0_NH4_	11.1 mol/m^3^	The initial concentration of ammonia ion
C_0_HPO4_	3.45 mol/m^3^	The initial concentration of phosphate ion
r_1_	9.58*10^−4^ mol/(m^3^·s)	The reaction rate of struvite precipitation
r_2_	1*10^−2^ mol/(m^3^·s)	Neutralization rate

## Supplementary information

Supplementary Information
