# Supplementary material for: Phosphate Recovery from Swine Wastewater by a Struvite Precipitation Electrolyzer
Source: Sci Rep. 2019 Jun 20;9:8893. doi: 10.1038/s41598-019-45085-3 (PMC6586645; doi:10.1038/s41598-019-45085-3)
Supplement: Supplementary file 1 — Supplementary Information [file 41598_2019_45085_MOESM1_ESM.pdf]

Supplementary data

Phosphate Recovery from Swine Wastewater by a Struvite Precipitation Electrolyzer

Fang Wang[1], Rao Fu[1], Hang Lv[1], Guoliang Zhu[2], Binwei Lu[1], Zheng Zhou[2], Xu Wu[1,*], Huanchun Chen[3]

1, School of Environmental Science and Engineering, Huazhong Unviersity of Science and Technology, 1037 Luoyu Road, Wuhan, 430074, China

2, Hubei Meichen Environmental Protection Science and Technology Co., Ltd., No. 6 Gaoxin Road, High-tech Zone, Jingmen, 448000, China

3, College of Animal Science and Technology, Huazhong Agricultural University, 1 Lion Rock, Wuhan, 430070, China

3.1. SEM-EDS pictures, particle size distribution and XRD patterns of the struvite precipitation at different reaction rate.

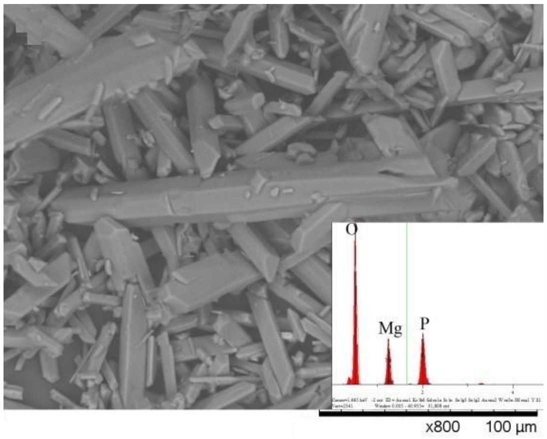

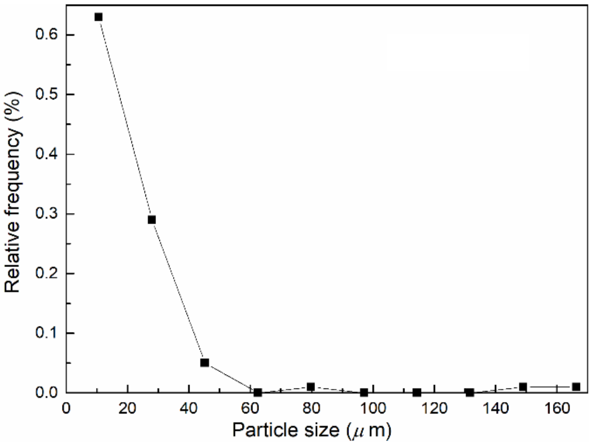

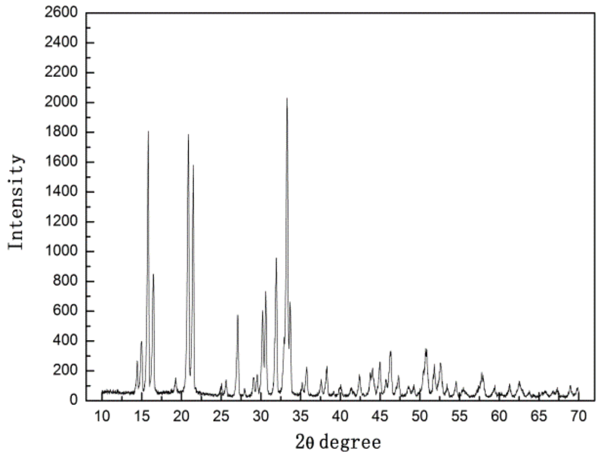

Figure S1. SEM-EDS pictures, particle size distribution and XRD patterns of the struvite precipitation at reaction rate of 70.46 mg/L·h, (phosphate concentration, 20 mg/L; pH, 8.6)

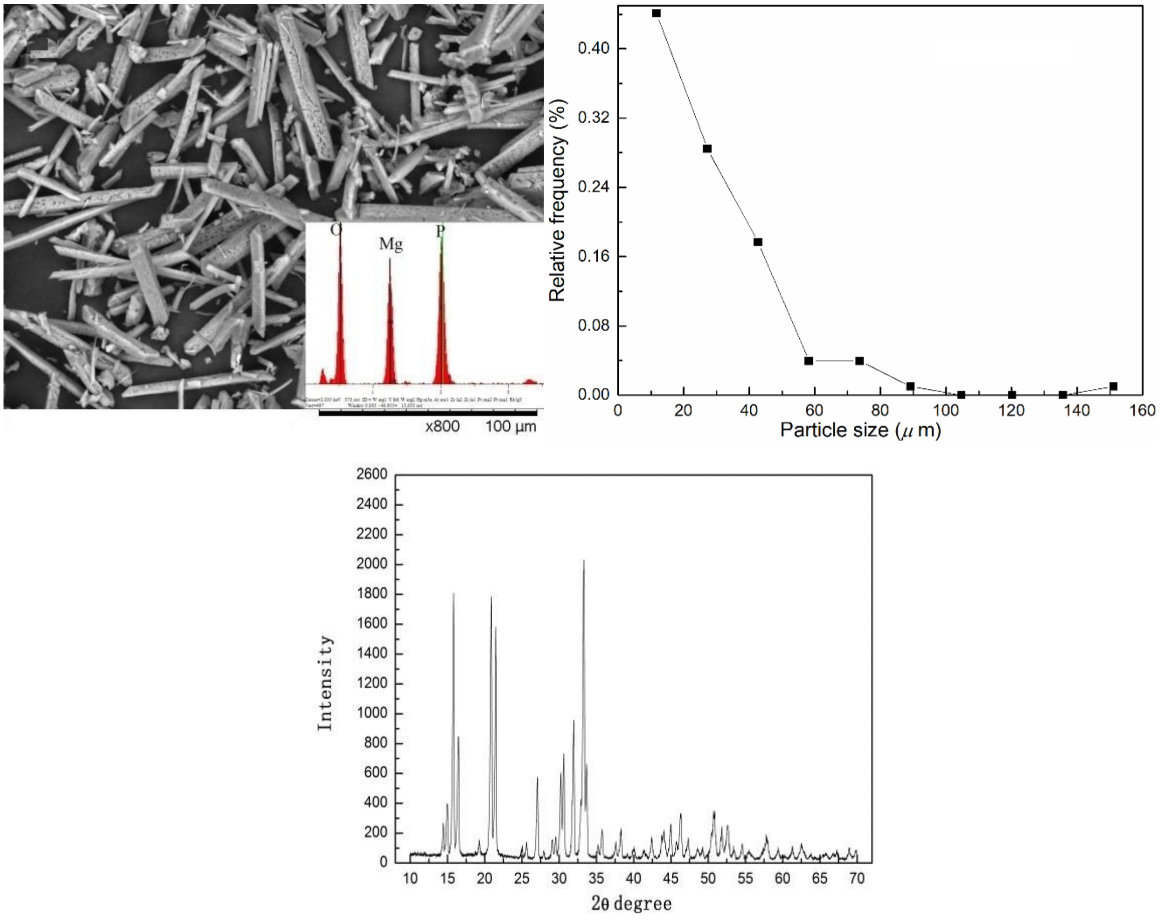

Figure S2. SEM-EDS pictures, particle size distribution and XRD patterns of the struvite precipitation at reaction rate of 150.55 mg/L·h, (phosphate concentration, 40 mg/L; pH, 8.57)

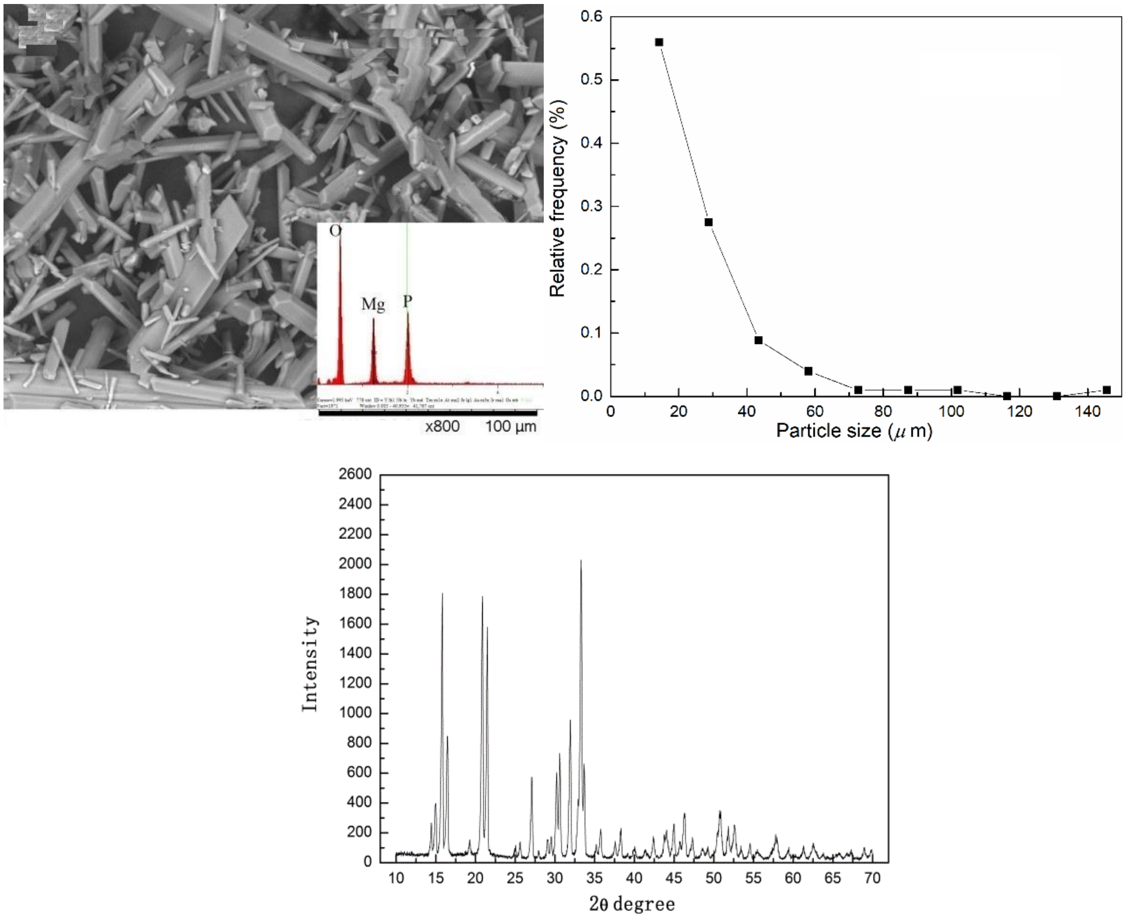

Figure S3. SEM-EDS pictures, particle size distribution and XRD patterns of the struvite precipitation at reaction rate of 210.87 mg/L·h, (phosphate concentration, 60 mg/L; pH, 8.74)

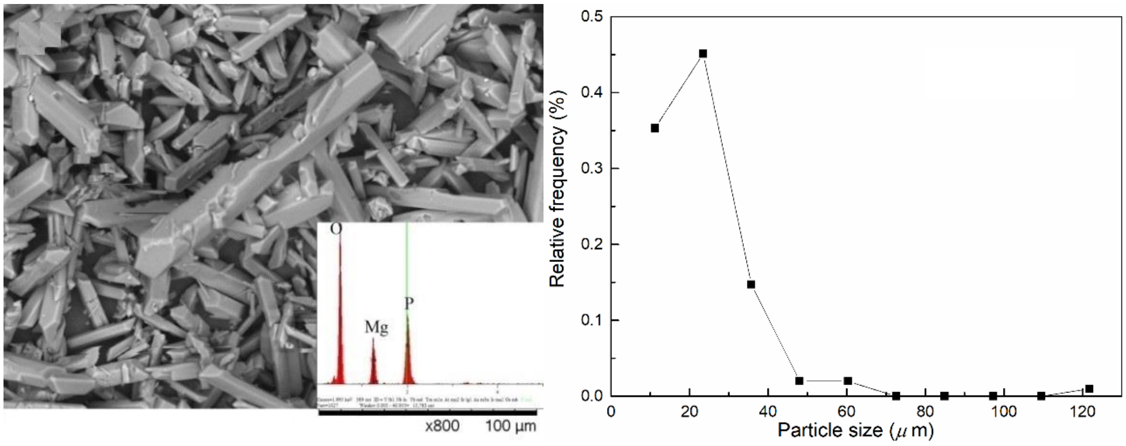

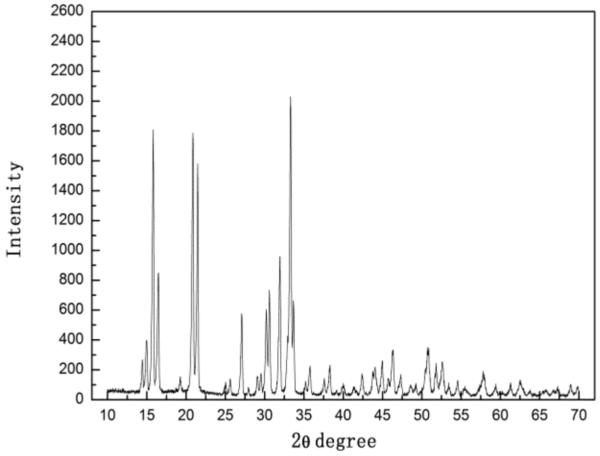

Figure S4. SEM-EDS pictures, particle size distribution and XRD patterns of the struvite precipitation at reaction rate of 311.31 mg/L·h, (phosphate concentration, 80 mg/L; pH, 8.97)

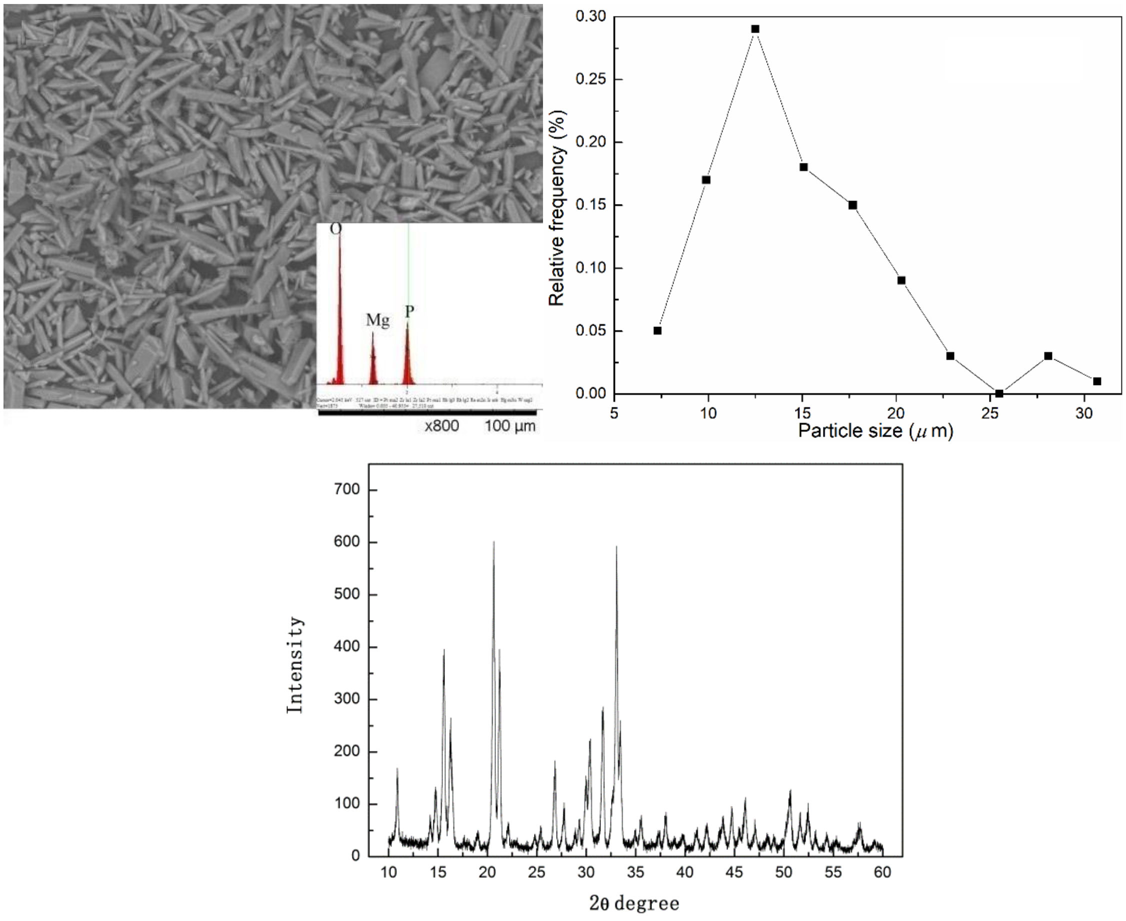

Figure S5. SEM-EDS pictures, particle size distribution and XRD patterns of the struvite precipitation at reaction rate of 396.65 mg/L·h, (phosphate concentration, 100 mg/L; pH, 9.08)

3.2.1 Numerical simulation results for the variation and distribution of pH in the

electrolyzer at different flow rates and currents.

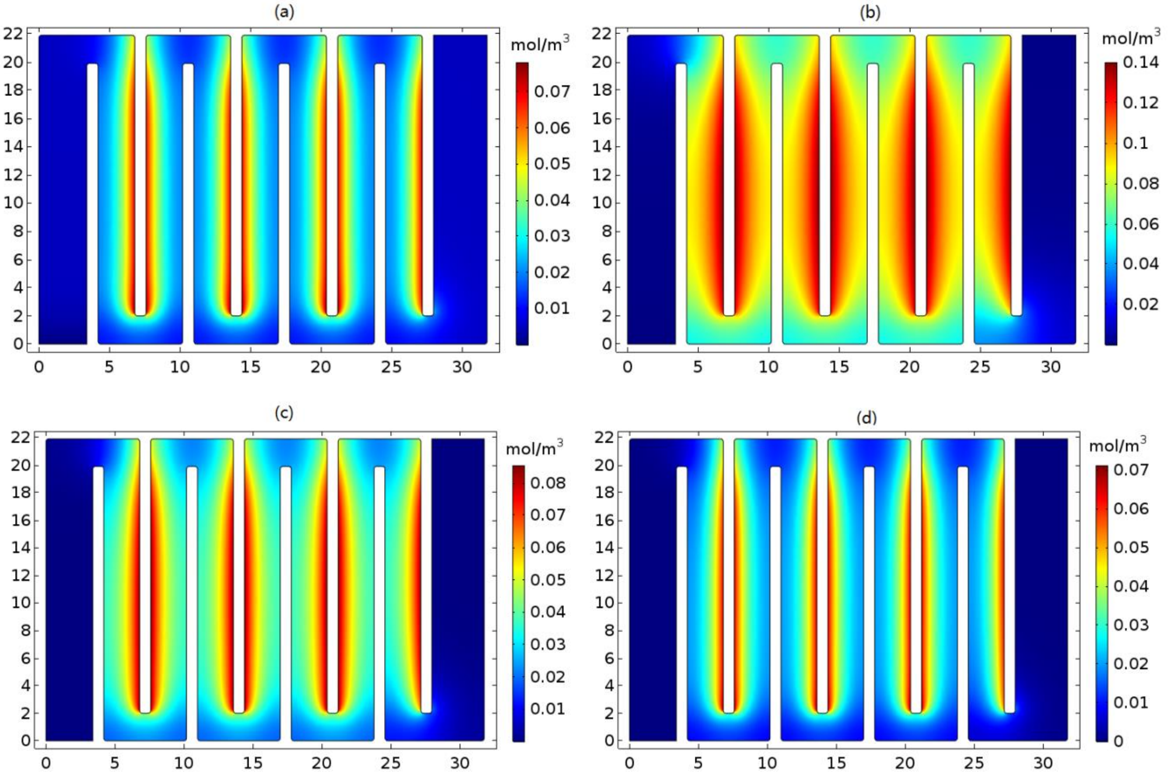

Figure S6. The model diagrams of variation and distribution of pH in the electrolyzer at the reaction time of (a) 100s, (b) 1000s, (c) 2500s and (d) 3700s. (Flow rate, 0 L/h; the current, 1.13 A)

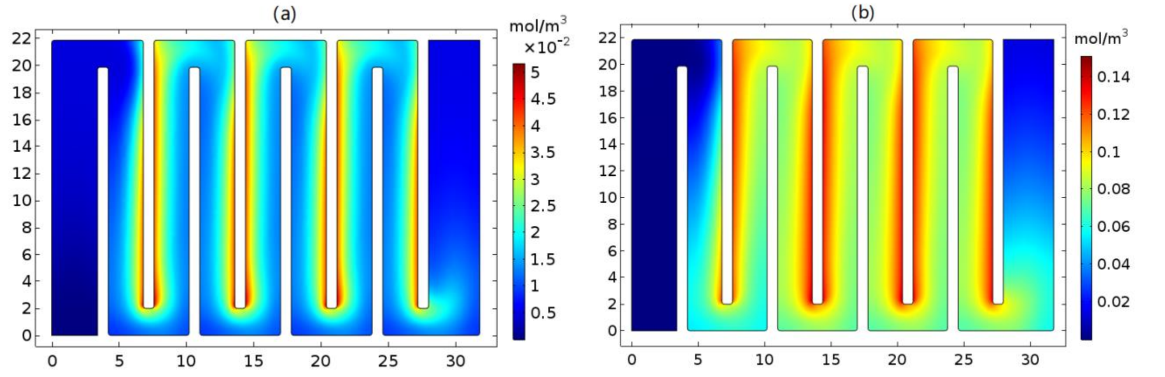

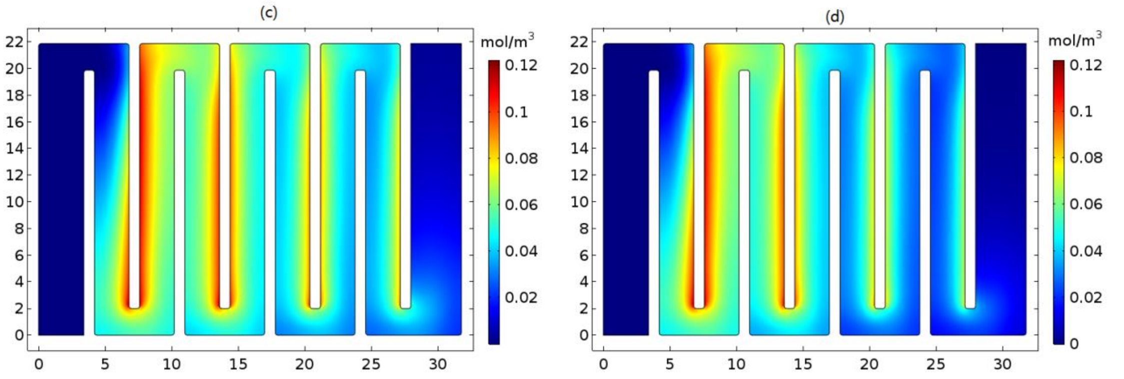

Figure S7. The model diagrams of variation and distribution of pH in the electrolyzer at the reaction time of (a) 100s, (b) 1000s, (c) 2500s and (d) 3700s. (Flow rate, 10 L/h; the current, 1.13 A)

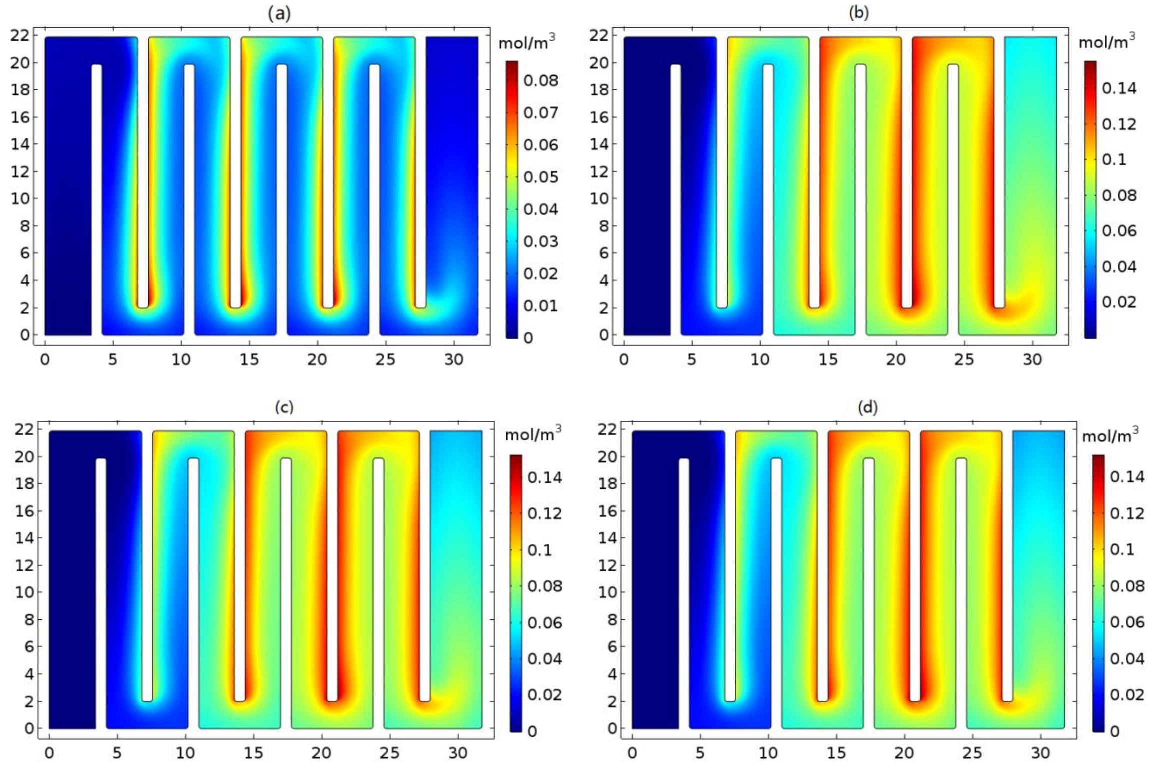

Figure S8. The model diagrams of variation and distribution of pH in the electrolyzer at the reaction time of (a) 100s, (b) 1000s, (c) 2500s and (d) 3700s. (Flow rate, 30 L/h; the current, 1.13 A)

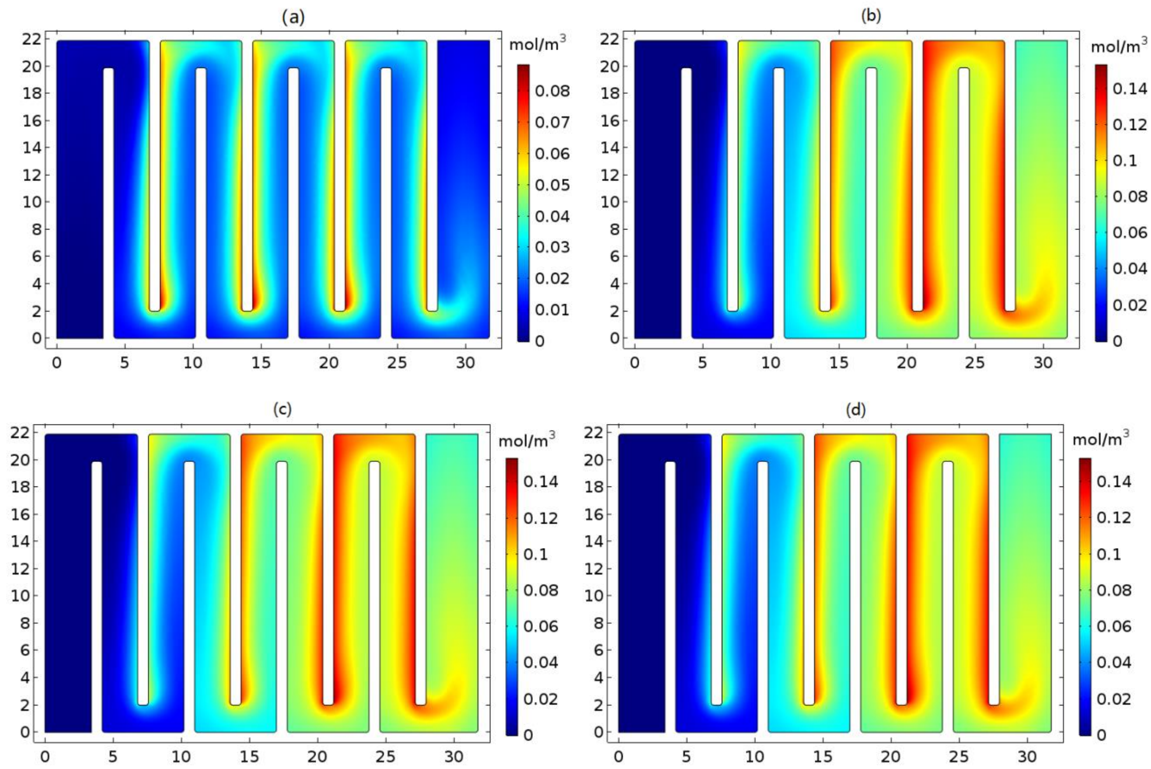

Figure S9. The model diagrams of variation and distribution of pH in the electrolyzer at the reaction time of (a) 100s, (b) 1000s, (c) 2500s and (d) 3700s. (Flow rate, 40 L/h; the current, 1.13 A)

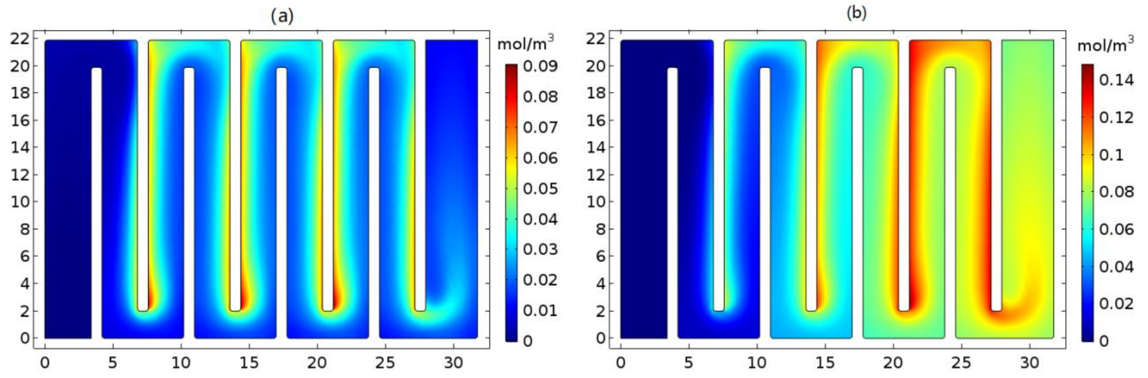

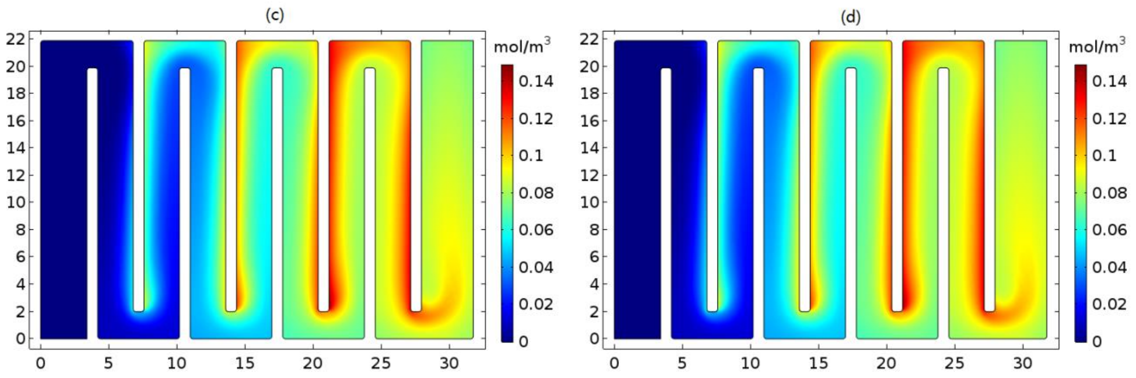

Figure S10. The model diagrams of variation and distribution of pH in the electrolyzer at the reaction time of (a) 100s, (b) 1000s, (c) 2500s and (d) 3700s. (Flow rate, 50 L/h; the current, 1.13 A)

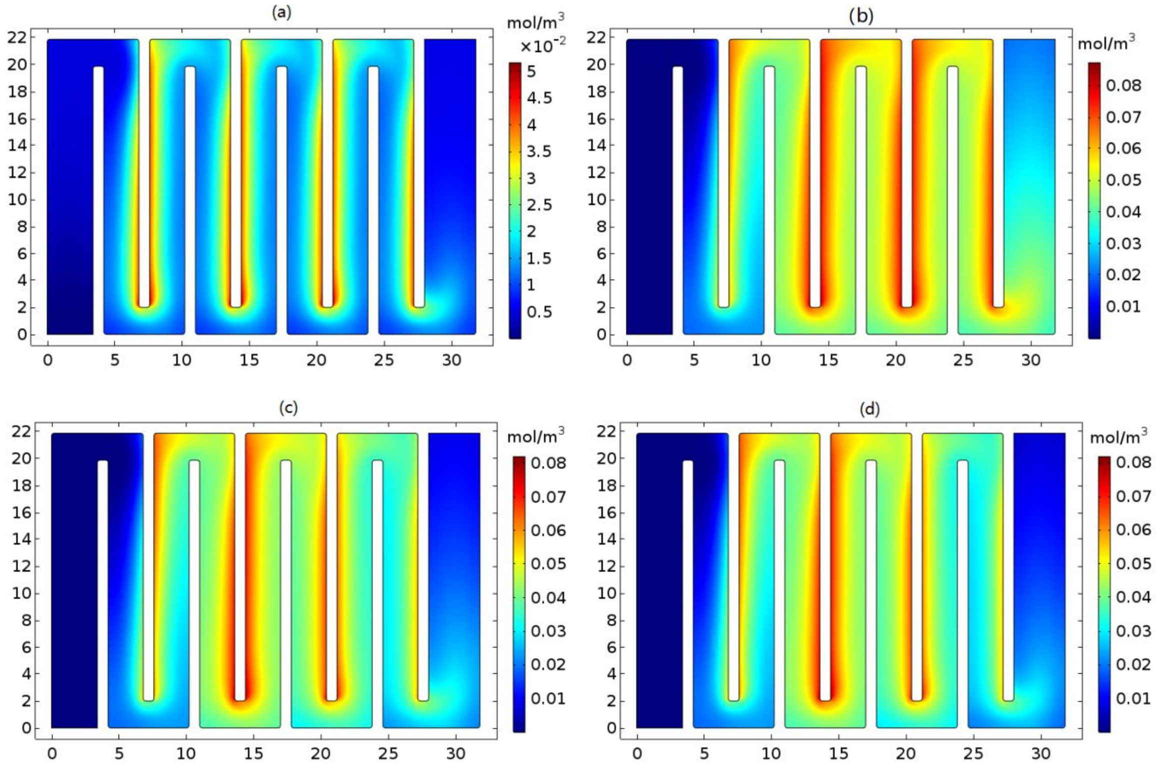

Figure S11. The model diagrams of variation and distribution of pH in the electrolyzer at the reaction time of (a) 100s, (b) 1000s, (c) 2500s and (d) 3700s. (Flow rate, 20 L/h; the current, 0.65 A)

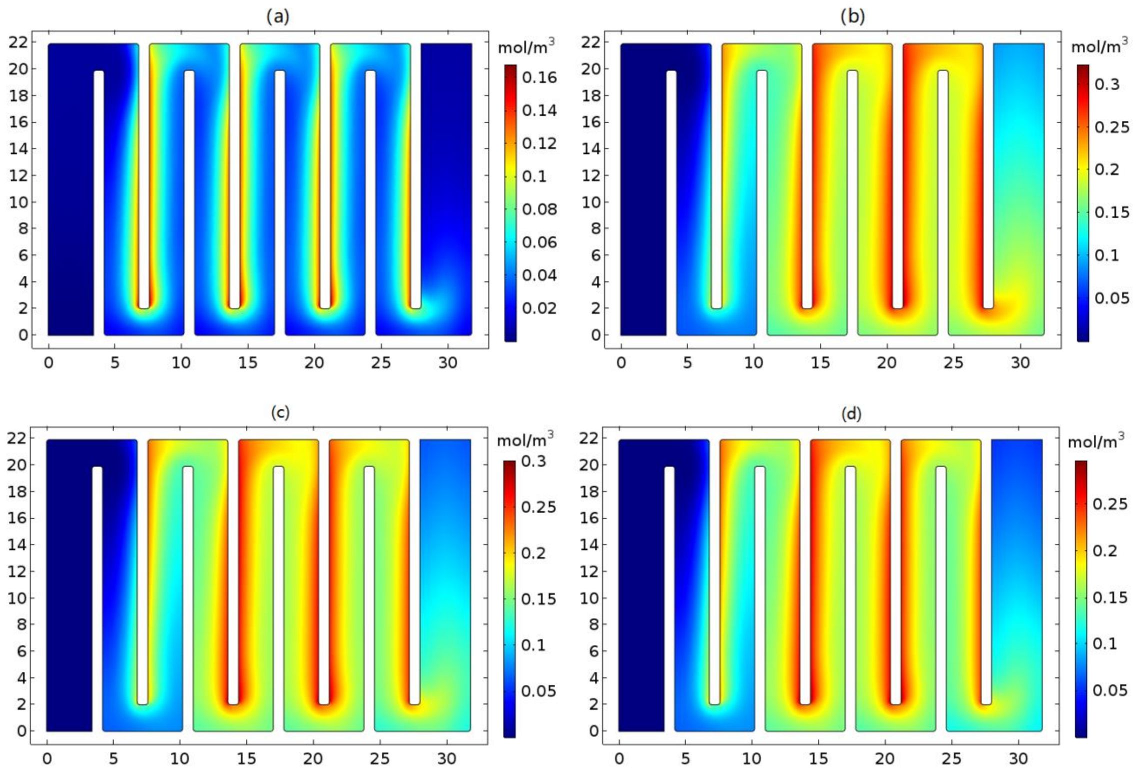

Figure S12. The model diagrams of variation and distribution of pH in the electrolyzer at the reaction time of (a) 100s, (b) 1000s, (c) 2500s and (d) 3700s. (Flow rate, 20 L/h; the current, 2.26 A)

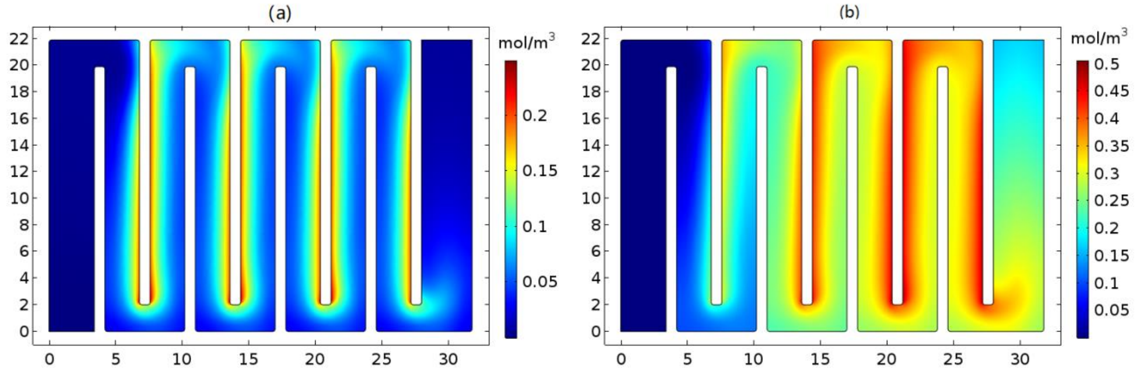

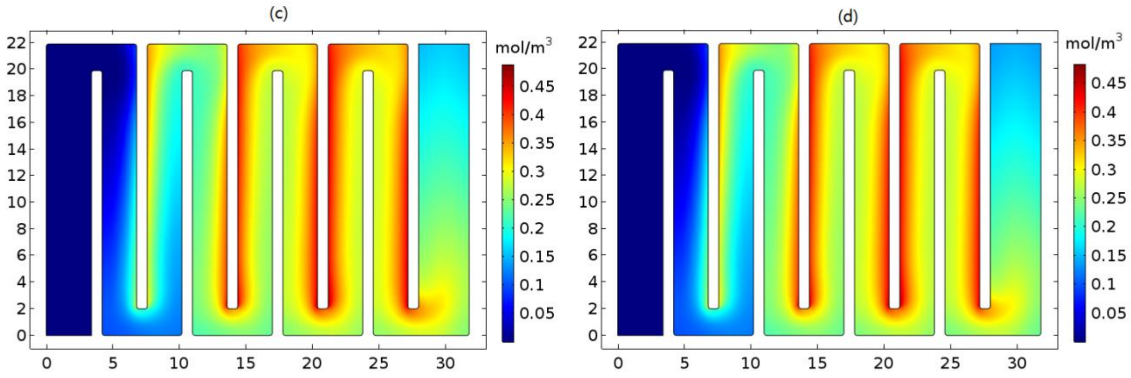

Figure S13. The model diagrams of variation and distribution of pH in the electrolyzer at the reaction time of (a) 100s, (b) 1000s, (c) 2500s and (d) 3700s. (Flow rate, 20 L/h; the current, 3.39 A)

3.2.2. Change of pH in the distribution point of flow field in the electrolyzer with time under different flow rates and currents.

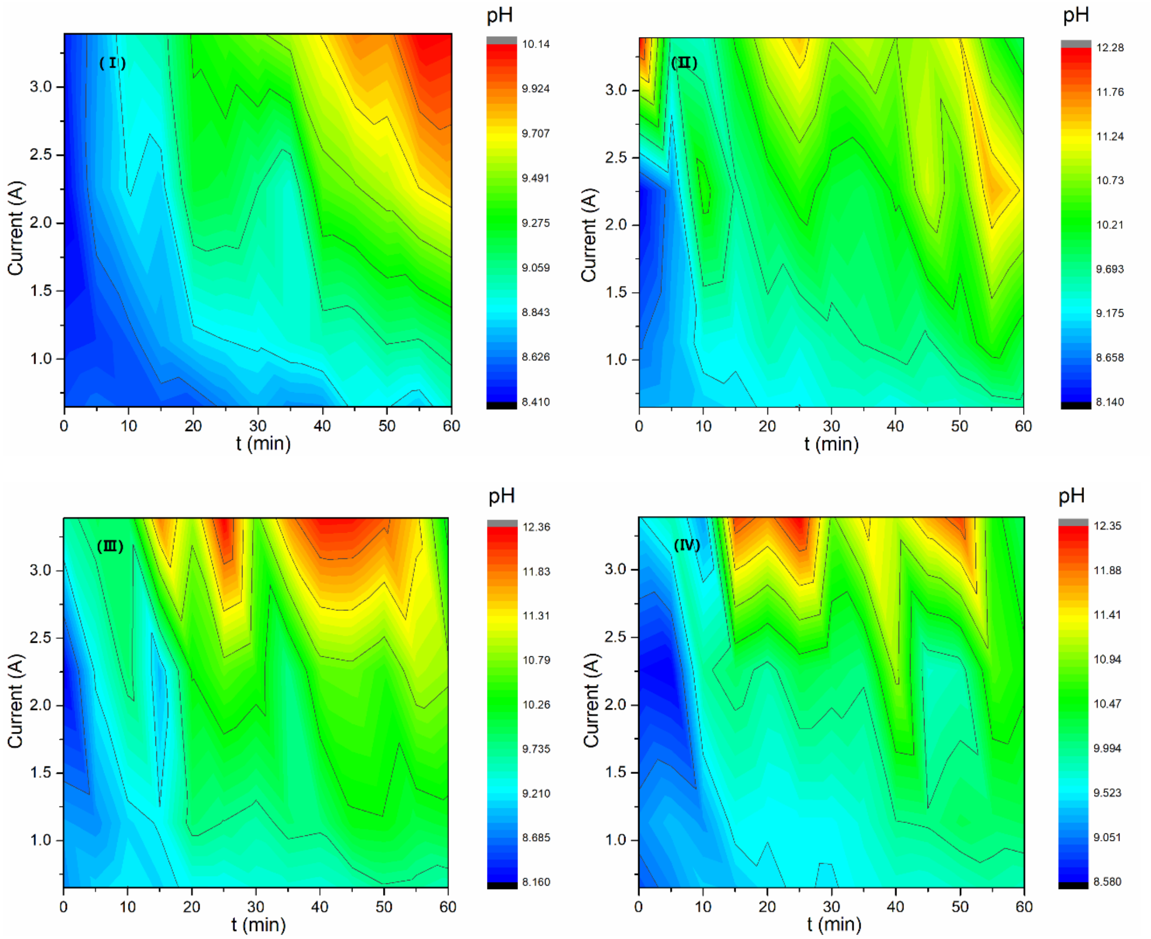

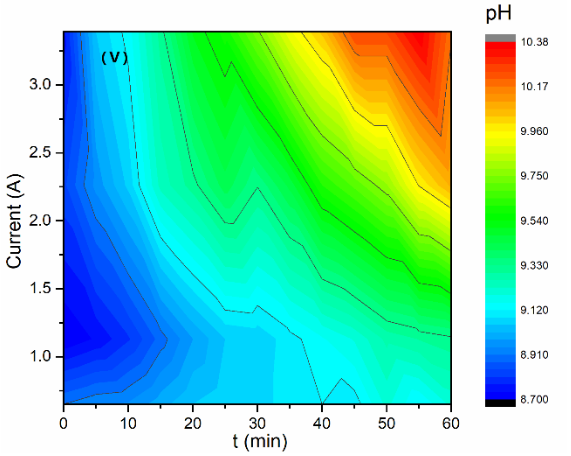

Figure S14. Under different current conditions, change of pH in the distribution point of flow field in the electrolyzer with time (flow rate, 0 L/h)

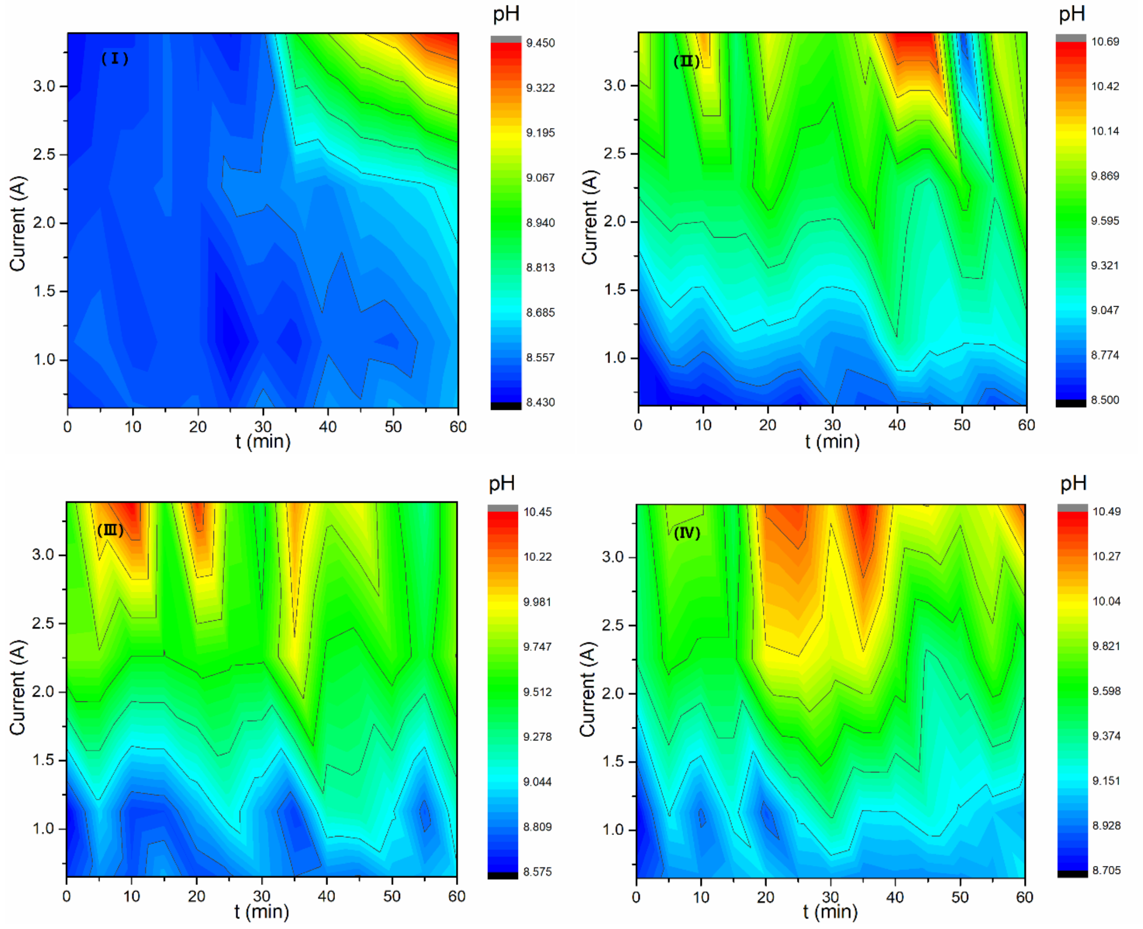

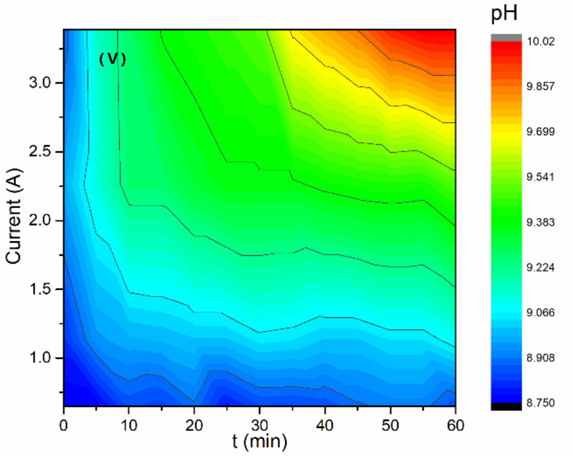

Figure S15. Under different current conditions, change of pH in the distribution point of flow field in the electrolyzer with time (flow rate, 10 L/h)

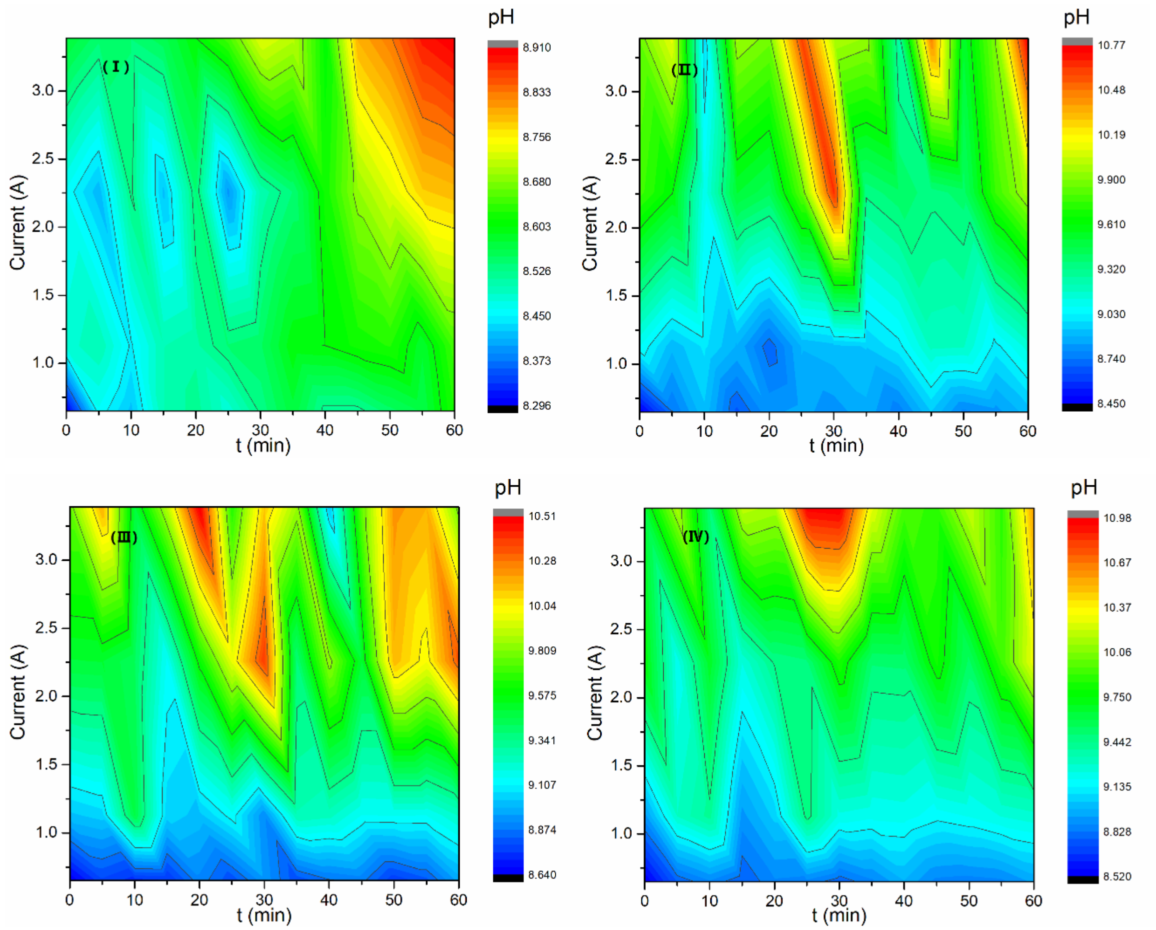

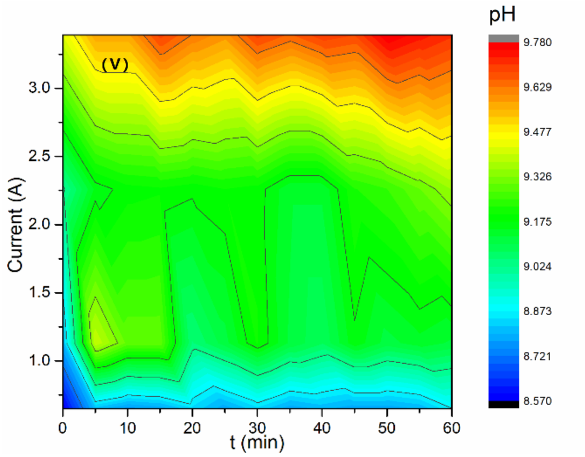

Figure S16. Under different current conditions, change of pH in the distribution point of flow field in the electrolyzer with time (flow rate, 20 L/h)

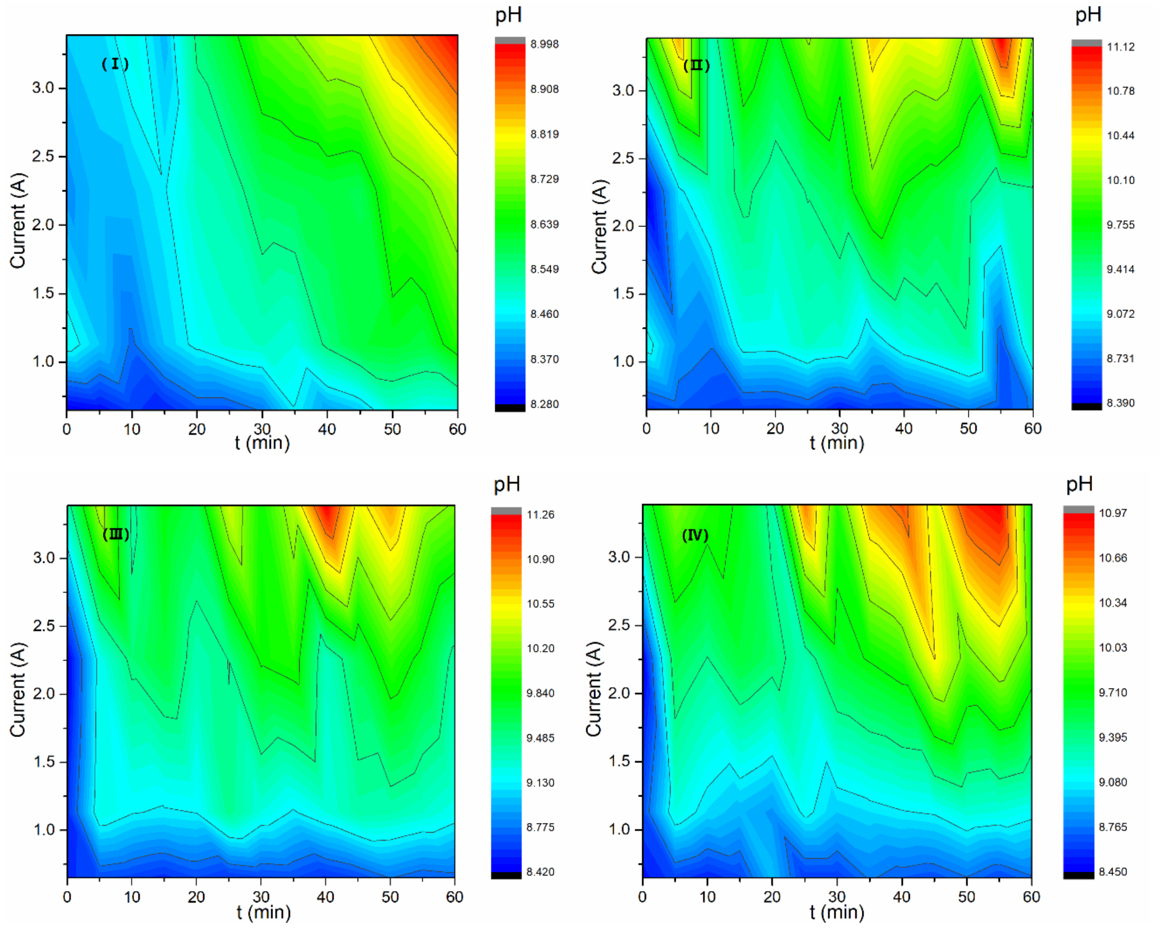

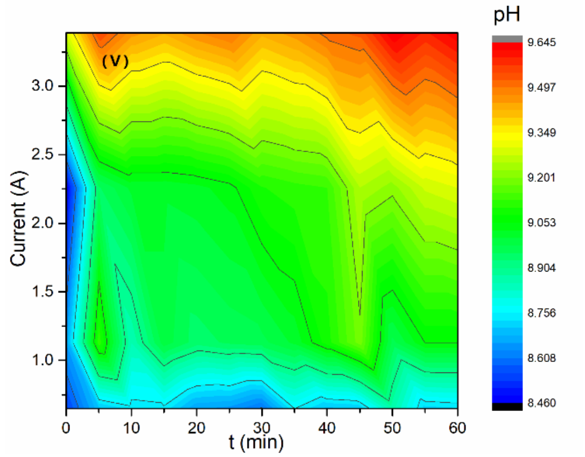

Figure S17. Under different current conditions, change of pH in the distribution point of flow field in the electrolyzer with time (flow rate, 30 L/h)

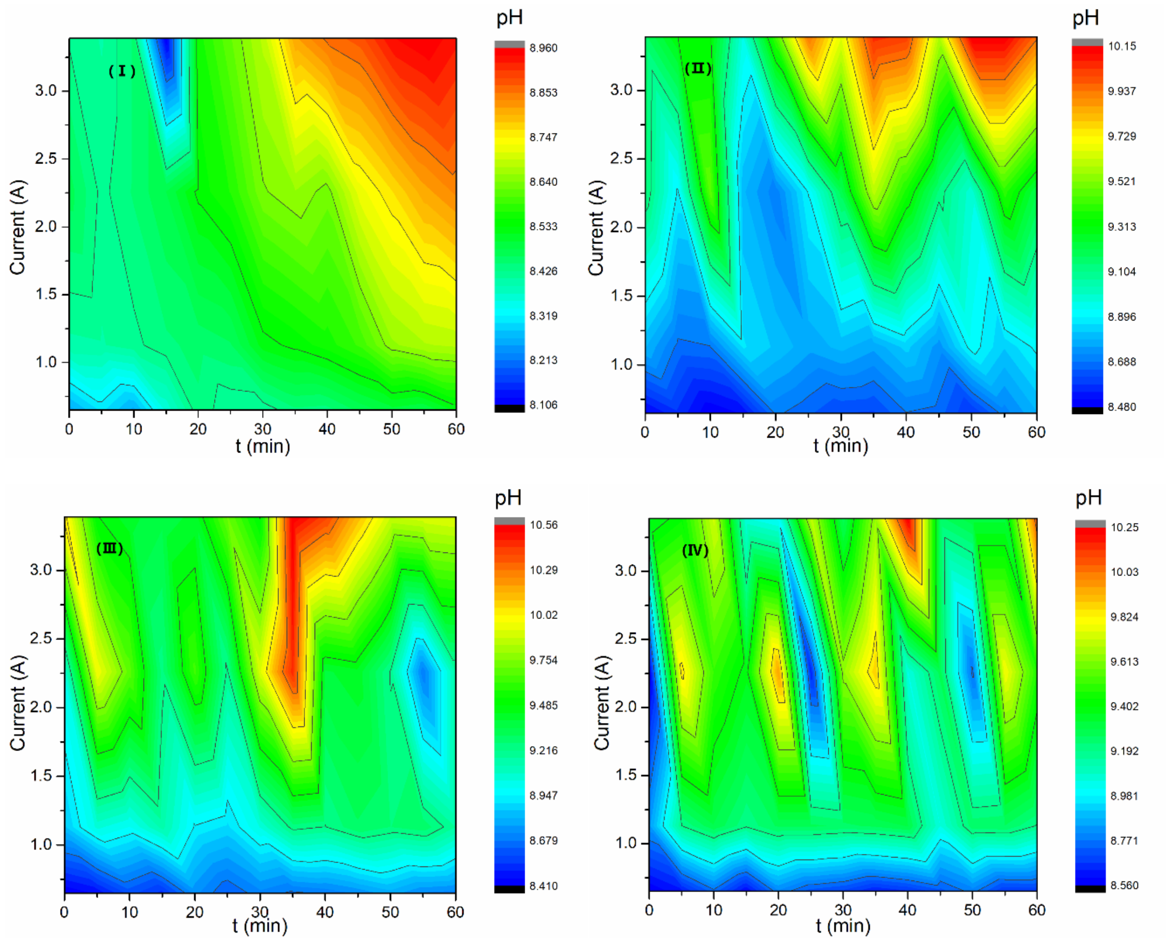

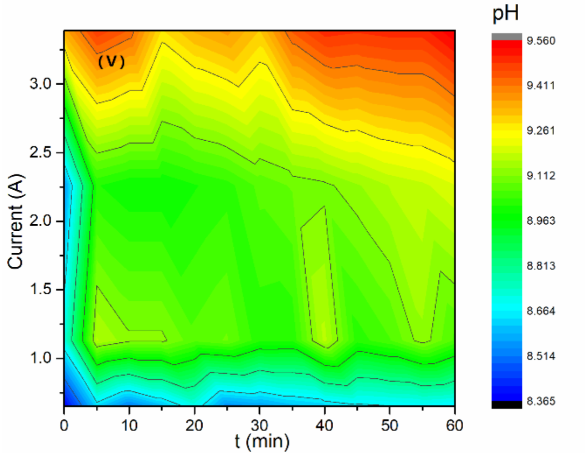

Figure S18. Under different current conditions, change of pH in the distribution point of flow field in the electrolyzer with time (flow rate, 40 L/h)

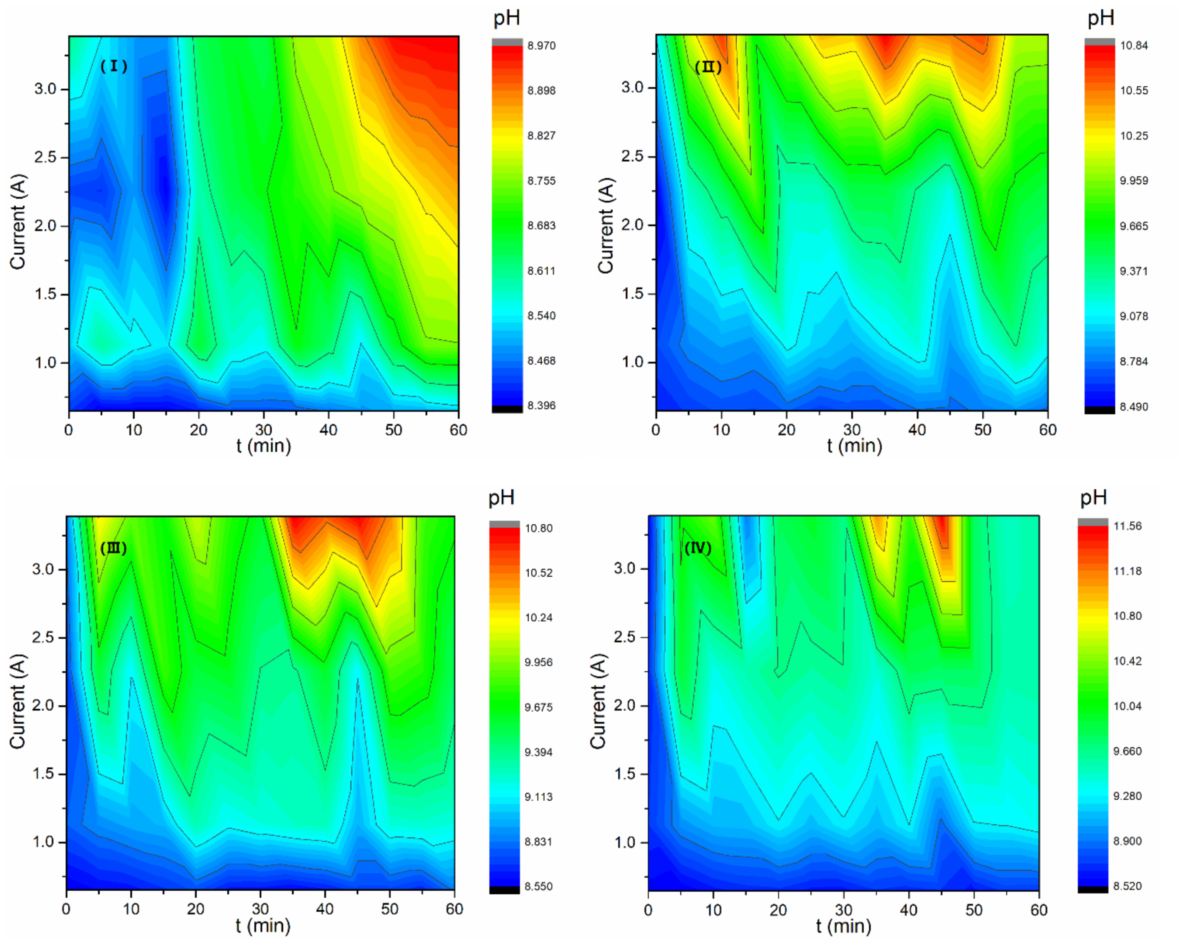

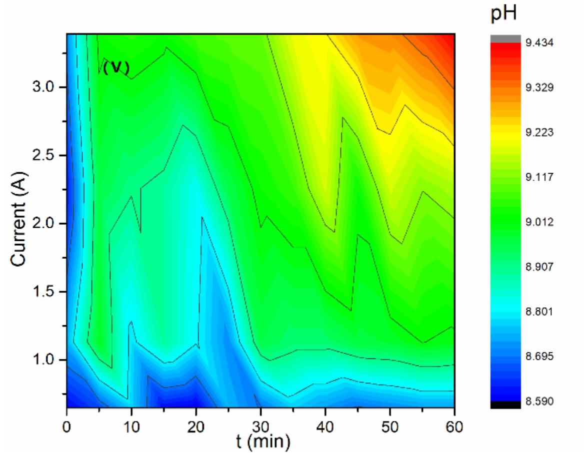

Figure S19. Under different current conditions, change of pH in the distribution point of flow field in the electrolyzer with time (flow rate, 50 L/h)

3.4. Energy consumption.

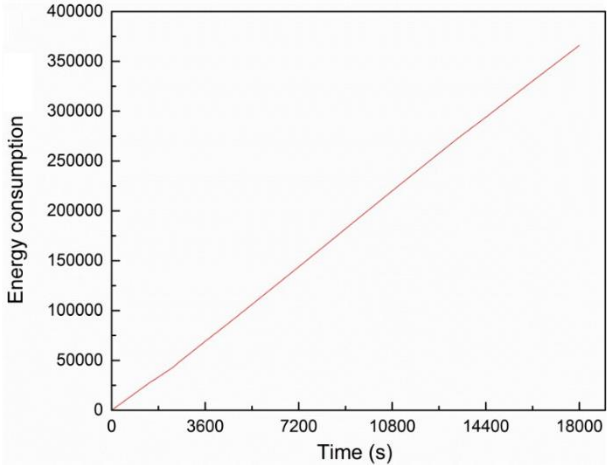

Figure S20. Energy consumption.
